# Hydrolytic stress degradation study and concomitant HPTLC estimation of thioctic acid and biotin in their combined capsules: greenness, blueness and whiteness assessment

**DOI:** 10.1186/s13065-025-01637-5

**Published:** 2025-10-29

**Authors:** Dina Salah El-Kafrawy, Amira H. Abo-Gharam

**Affiliations:** Pharmaceutical Chemistry Department, Faculty of Pharmacy, University of Alexandria, Elmessalah, 21521 Alexandria Egypt

**Keywords:** Thioctic acid, Biotin, Stability-indicating HPTLC, Degradation kinetics, Tri-faceted appraisal of sustainability

## Abstract

Stability testing and quality control assessment of active substances in single-pill combinations represent real challenges to drug analysts. This analytical challenge becomes more difficult when these drugs are non-chromophoric and co-formulated in disparate concentration ratio with few or no analytical methods reported in the literature for their concurrent quantification. This study deals with the development of HPTLC method for stability testing of hydrolytic degradation and simultaneous estimation of TH and BO in pure form, fresh and expired combined capsules. HPTLC separation was achieved using aluminium HPTLC plates precoated with silica gel 60 F-254 and mobile phase consisting of chloroform: methanol: ammonia (8.5:1.5:0.05, by volume). Scanning was performed at 215 nm. The designed method was validated as per the ICH guidelines. Excellent linearity was proved by high values of correlation coefficient not less than 0.99976 over concentration ranges of 2.5–30 and 2.5–20 µg/band for TH and BO, respectively. The limits of dectection were 0.58 and 0.33 µg/band while the limits of quantitation were 1.74 and 0.99 µg/band for TH and BO, respectively. The method proved to be precise, accurate and robust with values of RSD% and Er% not exceeding 2%. The designed method was found selective for the simultaneous estimation of BO and TH in pure form, in the presence of their degradation products, in valid and expired combined capsules. Both drugs were subjected to wet heat, acidic and basic hydrolysis. Both drugs were found liable to degradation (11–19%) under acidic (2.5 M HCl at room temperature for 30 min) and alkaline (3 M NaOH at room temperature for 30 min) hydrolytic conditions but they were found more stable under neutral hydrolytic conditions. Furthermore, Degradation kinetic study was accomplished to better understand the kinetics of their degradation under the employed stress conditions. Moreover, a tri-faceted assessment of the method’s degree of greenness (Eco-Scale score is 80, AGREE score is 0.72 and MoGAPI score is 76), blueness (BAGI score is 82.5) and whiteness (score is 92.2%) was performed.

**Supplementary Information:**

The online version contains supplementary material available at 10.1186/s13065-025-01637-5.

## Introduction

**Fig. 1:**
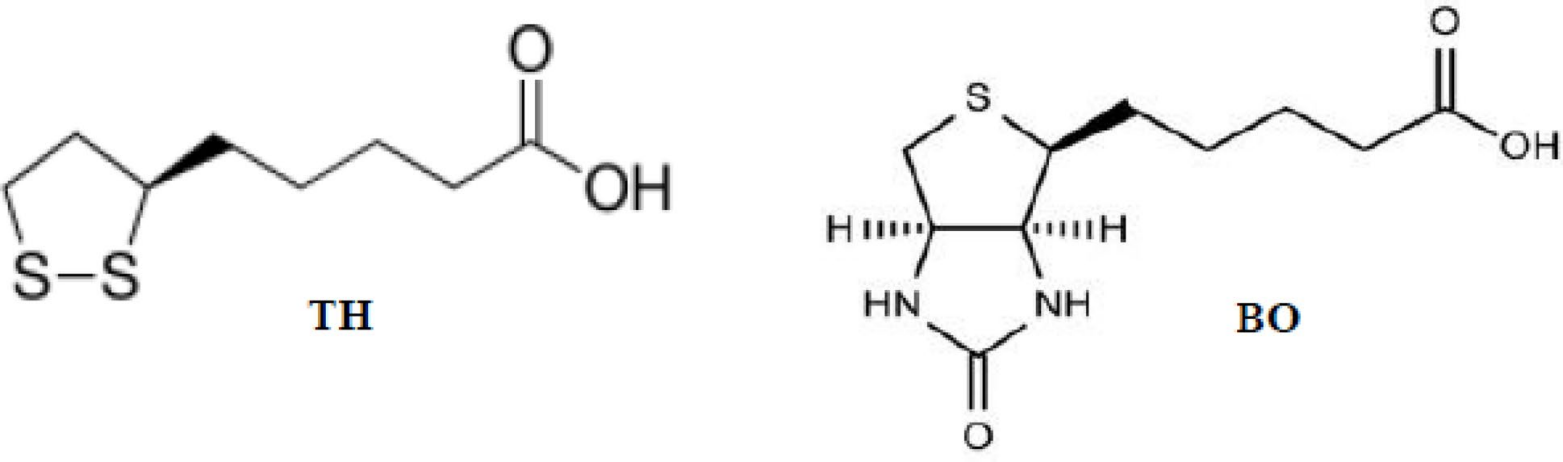
Chemical structures of thioctic acid (TH) and biotin (BO)

Thioctic acid [Alpha Lipoic acid] (1, 2-dithiolane-3-pentanoic acid) (TH) Fig. 1 is a natural antioxidant produced in human and animal cells, which has been administered as a dietary supplement possessing widespread therapeutic applications [1]. TH and its reduced metabolite [dihydrolipoic acid (DHLA)], scavenge a wide range of reactive oxygen radicals, regenerate endogenous antioxidants, possess metal chelating activity and restore oxidized proteins [2, 3]. Consequently, TH is commonly indicated for the management of neurodegeneration associated with diabetes, mitochondrial cytopathies, cardiovascular diseases, hepatitis, cataract, radiation damage and HIV infections [4–6]. Biotin (5-[(3aS,4 S,6aR)-2-oxohexahydro-1 H-thieno[3,4-d]imidazol-4-yl]pentanoic acid) (BO) (Fig. 1). Biotin is also known as vitamin B7 or coenzyme R, it is a coenzyme for carboxylase enzymes, thus it is necessary for cell growth, production of fatty acids, metabolism of fats and amino acids as well as in different metabolic reactions [7].

Supplemental BO is usually co-administered with TH products, especially at high doses of TH (>100 mg), because TH can compete with endogenous BO for binding to its transporter and decreases the cellular uptake of BO thus interferes with its normal biochemical activity in the body [8, 9]. Coformulation of BO with TH preparations is to provide a compensating role rather than a pharmacological effect. Based on the clinical importance of this combined formulation, it is crucial to establish robust and sensitive analytical methods for the concurrent determination and stability testing of both cited drugs.

Checking the scientific databases revealed many quantitative published studies applied HPLC methods with various detection modes for the determination of TH alone or with other drugs rather than BO [10–14]. HPTLC [15], Electrochemical [16–19], spectrophotometric [20, 21], spectrofluorimetric [22, 23], capillary electrophoresis [24], UPLC-MS/MS [25] and GC-MS [14] methods were also reported for TH determination. Similarly, many reported analytical methods applied HPLC technique with different detection modes for BO estimation alone or with other drugs rather than TH [26–29]. UPLC [30, 31], HPTLC [32, 33], MEKC [34], electrochemical [35] and spectrofluorimetric [36] methods were also published for BO determination.

Scientific database revealed only two ancient reports dealing with concurrent qualitative analysis of TH acid and BO with other components. The first report considers the concomitant qualitative analysis of thioctic acid biotin with their analogs and metabolites by high-performance liquid chromatography based on binding to avidin [37]. The second report concerns with the simultaneous qualitative detection of thioctic acid biotin with other water-soluble vitamins by RP-ion-interaction-reagent HPLC [38]. Both reported methods [37, 38] are regarded as screening qualitative methods rather than quantitative ones. Stability chromatographic studies were reported for TH either alone [39, 40] or concomitantly with other co-formulated drugs [41, 42], also another stability study was published for BO simultaneously with co-formulated viamins [43]. On the other hand, no analytical report is available in the literature for the simultaneous determination and stability testing of TH and BO. This encouraged us to develop a simple, accurate and reliable HPTLC method to afford a helpful analytical tool for the stability testing and concurrent determination of both analytes.

Due to the current lack of validated analytical methods capable of simultaneously quantifying and determining the stability of TH and BO under various stress conditions. This encouraged us to address this analytical gap by developing a novel stability-indicating and validated HPTLC method for simultaneous quantification of TH and BO in their combined pharmaceutical formulation. This study addresses the complexity involved in the simultaneous analysis of TH and BO due to the very minute concentration of the non chromogenic drug BO in Thioglu^®^ capsules at a ratio of 100: 1, TH: BO beside the close chemical similarity of both drugs which increases the difficulty in accomplishing their neat chromatographic separation. Also, the study of the inherent stability of both drugs was performed upon subjecting them to wet heat, acidic and alkaline hydrolysis with good resolution of the main drugs from their forced degradation products' peaks. The proposed method was also applied for the simultaneous determination of TH and BO in their valid Thioglu^®^ capsules without interference from existing excipients and for the analysis of expired Thioglu^®^ capsules. Additionally, the developed method achieved effective neat separation of the target drugs from the actually existing degradation products generated upon aging of the capsules as confirmed by chromatographic separation and peak purity analysis. Furthermore, degradation kinetic study was accomplished to better understand the kinetics of their degradation under the employed stress conditions.

Moreover, a tri-faceted assessment of sustainability was performed to check the method’s degree of greenness, blueness and whiteness using multiple complementary metrics. The most commonly applied Analytical Eco-Scale [44], AGREE (Analytical GREEnness) [45] and the recently endorsed MoGAPI (Modified Green Analytical Procedure Index—September 2024) [46] metrics are applied to assess and compare the method’s greenness.The BAGI (Blue applicability grade index) [47] metric and the RGB12 algorithm (Red-Green-Blue) [48] were applied to assess its applicability (blueness) and sustainability (whiteness), respectively. Thus, the proposed method could be employed as a sustainable and cost effective method alternative to more complex methods, making it convenient to quality control labs especially those with limited resources in manufacturing companies in developing countries.

## Experimental

### Instrumentation and software

The analytes were accurately weighed using a 4-digits Vibra electronic balance. The stock, degradation and dosage form solutions were dissolved and extracted using a sonicator water-bath. HPTLC instrument includes a CAMAG Hamilton micro-liter syringe (100 µL) under a nitrogen stream using a CAMAG Linomat IV sample applicator (Switzerland). Precoated HPTLC silica gel aluminum plates 60 F254 (20 × 10 cm, 200 μm thickness with fluorescent indicator at 254 nm, Merck, Darmstadt, Germany) were used. Development was performed in a CAMAG twin trough glass chamber (20 × 20 cm) saturated with the mobile phase. Densitometric scanning was performed on CAMAG TLC scanner III operated by CATS software (V 3.15 CAMAG). The source of radiation utilized was a deuterium lamp emitting a continuous UV spectrum between 190 and 400 nm. The evaluation of greenness was calculated and sketched using freely available softwares such as analytical GREEnness calculator (AGREE) (downloadable from https://mostwiedzy.pl/AGREE.) for AGREE metric and MoGAPI (https://fotouhmansour.github.io/MoGAPI/) software for MoGAPI tool. For the assessment of practicality and applicability, the online BAGI software (https://bagi-index.anvil.app/) was handled to calculate BAGI score. Additionally, the method’s whiteness was appraised using freely available Excel spreadsheet available at https://ars.els-cdn.com/content/image/1-s2.0-S0165993621000455-mmc2.xlsx.

### Materials

Thioctic acid 99.8% and biotin 99.6% pure standards were kindly gifted from Pharco Pharmaceuticals (Amriya, Alexandria, Egypt) and Medizen Pharmaceutical Industries (Borg El Arab City, 4th district, block 2, Alexandria, Egypt), respectively. For stability testing, the % purities of the investigated drugs were verified in accordance with their previously reported validated procedures [10, 27], and were found to be consistent with the values claimed by their manufacturers. HPLC grade methanol (Fisher Scientific, Loughborough, UK), analytical grade chloroform (Fisher Scientific, Loughborough, UK), Ammonia solution extra pure (33% NH_3_) (Honeywell Wunstorfer Germany), sodium hydroxide (El-Nasr Chemical Ind. Co., Egypt) and hydrochloric acid 37% (Merck, Darmstadt, Germany) were employed in this study. The pharmaceutical preparations used in this investigation were valid Thioglu^®^ capsules containing 300 mg TH and 3 mg BO per capsule BN 510591 and expired Thioglu^®^ capsules BN 410513 that were manufactured by (Arab Company for Pharmaceuticals and Medicinal Plants MEPACO-MEDIFOOD-Enshas El Raml-Sharkeya, Egypt).

### General procedures

#### Chromatographic conditions

CAMAG microlitre syringe was used for spotting 5 mm bands where all bands were spaced from each other by a distance of 4 mm on silica gel aluminum HPTLC plates 60 F254 (20 × 10 cm) leaving 10 mm distance from the bottom and 7 mm from the start side of the plate (Figure S1 in supplementary file 1). The solvent system consisted of chloroform–methanol–ammonia (8.5:1.5:0.05, by volume), the chamber saturation time was 20 min. The length of chromatogram run was 8.5 cm. CAMAG TLC scanner III was employed in the absorbance mode at 215 nm for scanning of developed HPTLC plates. The HPTLC scanner was calibrated. The slit dimensions were set to 4.00 × 0.30 mm, Micro, scanning speed was optimized to 20 mm/s and detection threshold was adjusted based on preliminary trials to maximize signal to noise ratio and resolution. The laboratory temperature was maintained constant at 25 ± 1 °C. Regarding the humidity, the lab was air-conditioned which helps to regulate humidity. The mentioned chromatographic conditions were selected following a series of optimization trials involving various mobile phase combinations, saturation times, and detection wavelengths. The complete optimization process is presented in “Results and discussion” section.

#### Construction of calibration graphs

Standard solutions of thioctic acid and biotin were prepared in methanol to contain 4 mg/mL and 2.5 mg/mL, respectively. In 5 mL volumetric flasks, working ranges were prepared by dilution of aliquots of the stock solutions with methanol to have final concentration ranges of 0.25–3 mg/mL for TH and 0.25–2 mg/mL for BO which were found lower than the marketed concentrations for both drugs. Triplicate spotting of 10 µL of each concentration on HPTLC plates was performed. After air drying of bands, the HPTLC plates were developed by applying ascending development technique and the previously described chromatographic conditions.

Noteworthy, prior to samples spotting, HPTLC plates were activated by preheating the plates in thermostatically controlled oven at 100 °C for 20 min.

#### Application to pharmaceutical formulation

The content of twenty valid Thioglu^®^ capsules was carefully mixed, weighed and ground. For BO content determination, a weight equivalent to 50 mg BO was extracted with 30 mL methanol by sonnication for 20 min then filtered into 50 mL volumetric flask. The residue was washed with 2 × 5 mL portions of methanol, washings were added to the filtrate and finally the solution was completed to volume with the same solvent and assayed as under “Construction of calibration graphs”. For TH content determination, 5 mL of the prepared extract were diluted to 250 mL with methanol and assayed as under “Construction of calibration graphs”. Recovered concentrations were calculated from the corresponding external standards (simultaneously treated standard solution of the analyzed drugs).

For the standard addition assay, Aliquots of each sample solutions were spiked with aliquots of standard solutions of the analyzed drugs to obtain total concentrations within the specified ranges then treated as previously described. Recovered concentrations were calculated by comparing the analyte response with the increment response attained after addition of the standard.

Aged Thioglu^®^ capsules (about 3 years beyond expiry) was similarly extracted and analyzed as under “Construction of calibration graphs”. Recovered concentrations were calculated from the corresponding external standards (simultaneously treated standard solution).

For all performed previous studies on both fresh and aged formulations, 5 replicate spotting of 10 µL of each prepared sample solution was carried out and recovered concentrations were calculated.

#### Preparation of forced-degradation solutions

##### Wet heat degradation

In 10 mL volumetric flasks, 10 mg of TH or BO separately were dissolved in 2 mL methanol. Three milliliters of water were added. The solutions were kept in boiling water bath protected from light for 5 h with continuous compensation of volume with methanol, then they were cooled and diluted to volume with methanol to reach a final concentration of 1 mg/mL of TH and BO, separately. Third solution containing a mixture of 1 mg/mL of both TH and BO was treated with the same conditions.

##### Acidic hydrolytic degradation

In 10 mL volumetric flasks, 10 mg of TH or BO separately were dissolved in 2 mL methanol. Two milliliters of 2.5 M HCl were added. The solutions were kept at room temperature for 30 min protected from light. The degraded solutions were neutralized with NaOH and diluted to mark with methanol to reach a final concentration of 1 mg/mL of TH and BO, separately. Third solution containing a mixture of 1 mg/mL of both TH and BO was treated with the same conditions.

##### Basic hydrolytic degradation

In 10 mL volumetric flasks, 10 mg of TH or BO separately were dissolved in 4 mL 3 M NaOH. The solutions were set aside at room temperature for 30 min protected from light. The degraded solutions were then neutralized with HCl and diluted to mark with methanol to reach a final concentration of 1 mg/mL of TH and BO, separately. Third solution containing a mixture of 1 mg/mL of both TH and BO was treated with the same conditions.

All stress degraded solutions were filtered through 0.45 μm filter, A volume of 15 µL of each degradation flask was triplically spotted on the TLC plate to obtain a final concentrations of 15 µg/spot. Then, they were assayed as under “Construction of calibration graphs”. The densitograms of the degradation solutions were compared to its corresponding standard solutions to evaluate the decrease in the drug peak area to compute % degradation or to observe the appearance of any new degradation peaks.

#### Degradation kinetic studies

##### Acidic degradation

10 mg of TH or BO separately were dissolved in 2 mL methanol in a series of 10 mL volumetric flasks. Two milliliters of different concentrations of HCl solutions (1.0, 2.5, 5.0 M) were separately added in each flask. The solutions were left at room temperature for different periods of time (0.25, 0.50, 0.75, 1.00 h.). Then, each flask was separately neutralized to pH 7.0 with its corresponding concentration of NaOH to obtain a final concentration of 1 mg/mL of TH or BO, separately.

##### Basic degradation

Into a series of 10 mL volumetric flasks, 10 mg of TH or BO separately were subjected to 4 mL volumes of different concentrations of NaOH solutions (1.5, 3.0, 6.0 M) at room temperature. At different time intervals (0.25, 0.50, 0.75, 1.00 h), each flask was neutralized to pH 7.0 with its corresponding concentration of HCl to get a final concentration of 1 mg/mL of TH or BO, separately.

All stress degraded solutions were filtered through 0.45 μm filter, spotted in triplicates and assayed as under “Construction of calibration graphs”. The percentage of degradation was determined by calculating the decrease in the cited drug peak area in each analyzed sample compared to its standard densitogram.

## Results and discussion

High-Performance Thin-Layer Chromatography (HPTLC) is an advanced evolution of traditional thin-layer chromatography (TLC), offering superior separation, sensitivity, and resolution. It operates on the same fundamental principles as TLC but uses enhanced instrumentation, such as precise sample applicators, temperature-regulated development chambers, and scanning densitometers. A notable distinction lies in the use of high-quality silica gel 60 plates with finer particle sizes, which promote denser packing and smoother surfaces resulting in minimum band diffusion and sharp compact spots. Key advantages of HPTLC over conventional TLC include higher resolution, faster analysis time, improved sensitivity, reduced solvent use, reliable quantification, compatibility with various detection methods (e.g., UV, fluorescence, chemiluminescence and mass spectrometry), and better reproducibility through automation [49]. While HPLC remains the benchmark in pharmaceutical quality control, HPTLC has recently emerged as a promising alternative due to its eco-friendliness, cost-effectiveness, and operational simplicity. Its key advantages over HPLC include faster analysis, lower solvent use, minimal waste volume, minimal sample preparation, the ability to handle complex and crude mixtures, and simultaneous parallel sample analysis on a single plate rather than sequential way, multi-sample processing. The integration of HPTLC with advanced detectors like MS, FTIR, and laser spectroscopy has broadened its applications [50].

### Optimization of chromatographic conditions

**Fig. 2:**
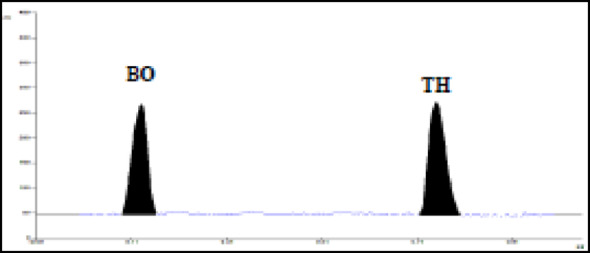
Representative densitogram of a mixture of TH (20 µg/band) and BO (20 µg/band)

Different organic non polar solvents such as: ethylacetate, n-hexane, dichloromethane (methylene chloride) and chloroform were tried mixed with polar modifiers such as ethanol or methanol. Only the mixture of chloroform and methanol afforded the best resolution of the cited drugs from their degradation products, but the developed peaks suffered from distorted shapes and broadness. Consequently, we tried the addition of either acetic acid or ammonia (pH modifiers) in varying proportions, only ammonia provided better symmetry and resolution for the developed peaks of both drugs from their degradation products. Finally, the most appropriate mobile phase combination was found chloroform: methanol: ammonia (8.5:1.5:0.05, by volume), this mixture afforded the best symmetry and acceptable resolution of the two main drugs from their degradation product peaks (as revealed from acceptable system suitability parameters mentioned below). The bands developed were dense and compact free from tailing or fronting effects with acceptable R_f_ values for TH (R_f_ = 0.75 ± 0.02) and BO (R_f_ = 0.12 ± 0.02) (Fig. 2). The impact of plate pre-treatment on chromatographic performance was systematically investigated to maximize separation efficiency and ensure reproducibility. Pre-activation of HPTLC plates prior to sample application, achieved by preheating in a thermostatically controlled oven, markedly reduced baseline noise and eliminated potential interferences arising from adsorbed impurities [51]. This procedure also enhanced spot symmetry and minimized tailing, without influencing peak areas or R_f_ values, as demonstrated in Figure S2 (Supplementary File 1).

Optimal activation was achieved by heating the plates at 100 ^∘^C for 20 min, whereas extending the heating time to 30 min or increasing the temperature to 120 ^∘^C did not yield further improvement in densitographic profiles. Following activation, samples were uniformly applied as 5 mm-wide bands using a CAMAG microlitre syringe, maintaining an inter-band spacing of 4 mm on silica gel 60 F254 aluminum plates (20 × 10 cm). Bands were positioned 10 mm above the lower edge and 7 mm from the side edge to minimize edge effects, prevent zone overlap, and ensure consistent solvent front development, thereby supporting high-resolution, reproducible separations. System suitability parameters were calculated for the HPTLC method, the resolution (R_s_) was found 9.86 ± 0.02, peak symmetry values were found 0.91 for TH and 0.96 for BO, tailing factors were found 0.92 for TH and 1.00 for BO, capacity factors (k’) were found 0.33 for TH and 7.33 for BO and finally the selectivity (α) value was found 22.21.These values were found within the recommended acceptance criteria [52] (Table S1 in supplementary file 1).

The UV absorption spectra of TH and BO show maximum absorption at 222 and 202 nm for TH and BO, respectively (Figure S3) (see Supplementary File 1) thus 215 nm was selected as the scanning wavelength to afford high sensitivity with minimum baseline noise especially for BO determination which exists in very low concentration ratio in the marketed combined formulation. HPTLC scanner helps in assessing peak purity of both analytes, especially in stress degraded sample solutions and extracted valid and aged dosage forms solutions.

#### Forced degradation studies

Various hydrolytic degradation conditions were applied using wet heat (water: methanol), acidic (2.5 M HCl) and strong basic (3 M NaOH) were carried out.

**Fig. 3:**
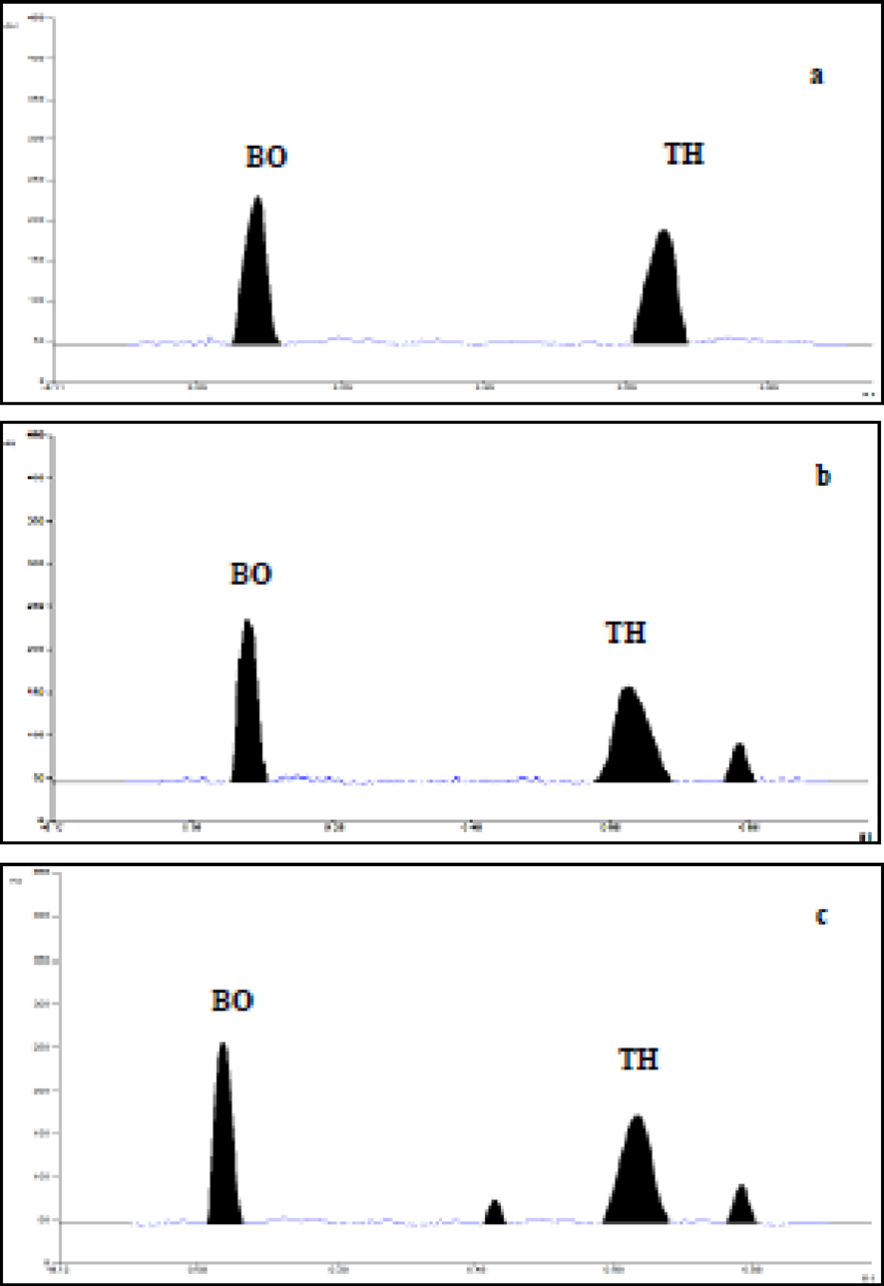
Densitograms of mixture of TH (15 µg/band) and BO (15 µg/band) after wet heat (**a**), acidic (**b**) and basic (**c**) hydrolysis

TH appeared to be stable under neutral hydrolytic condition after heating for 5 h at 100 °C in methanol (97.4% of intact TH was recovered) (Fig. 3a). Conversely, the drug is more sensitive towards acidic hydrolysis and loses ~ 19% of its potency with no appearance of any degradation product peak in HPTLC densitogram (Fig. 3b and Figure S4a in supplementary file 1). Under alkaline degradation conditions, 14% degradation occurred with the appearance of well resolved degradation product peak at R_f_ value 0.53 (Fig. 3c and Figure S4b in supplementary file 1). Regarding TH, our degradation study results were found consistent with previously reported methods [39–42]. For neutral degradation, TH was found stable as the published method [39]. Moreover, TH was found unstable under both acidic and basic hydrolysis where TH was more labile for acids than bases as revealed from our method and other reported ones [39–41]. Upon surveying the literature for the possible degradation pathway in alkaline medium, it is found that the strained five-membered 1,2-dithiolane ring, containing disulfide bond, is susceptible to nucleophilic attack by hydroxide ions [53, 54]. This leads to ring opening, forming dihydrolipoic acid or other thiol-containing intermediates. Therefore, the degradation product appeared at R_f_ 0.53 may be dihydrolipoic acid or other thiol-containing intermediates, but there is a need for further structure elucidation to confirm its identity in future lab work.

For BO, minute degradation (~ 4%) was observed after heating for 5 h at 100 °C in methanol with no detection of degradation product peaks (Fig. 3a). On the other hand, about 11% degradation occurred under acidic degradation conditions with elution of only one degradation product peak at 0.86 R_f_ value (Fig. 3b and Figure S4c in supplementary file 1). Under strong alkaline degradation condition, about 15–18% degradation occurred with the appearance of the same well resolved degradation product peak at 0.86 R_f_ value (Fig. 3c and Figure S4d in supplementary file 1). Different results of stress degradation study on the cited analytes are presented in Table S2 (supplementary file 1). Concerning BO, reviewing the literature illustrated only one reported stability indicating method for BO with other vitamins [43]. Relating the degradation results of the published method with our proposed one, BO is found unstable under both acidic and basic hydrolysis with nearly comparable degradation%. Reviewing the literature for common feasible pathway for acidic and basic degradation of BO, a review article mentioned that BO is degraded in strong acid or alkaline conditions and upon exposure to ultraviolet radiation, but the degradation mechanism and subsequent products have not been determined [55]. From chemistry point of view, BO Contains a tetrahydrothiophene ring fused to a ureido ring, and this ureido ring is a cyclic urea structure which is structurally like β-lactam ring in terms of strain and reactivity. The common degradation pathway of β-lactam antibiotics under both acidic and basic conditions is hydrolysis of the β-lactam ring, leading to ring-opened, inactive products such as penilloic acid [56]. Concerning BO, the probable common degradation hypothesis may target the ureido ring and break the C–N bonds in the ring, leading to ureido ring opening and producing urea derivatives or opened-chain biotin fragments. Actually, further structure elucidation is recommended in future lab work to confirm the identity of the formed degradation product.

#### Degradation kinetic studies

**Fig. 4:**
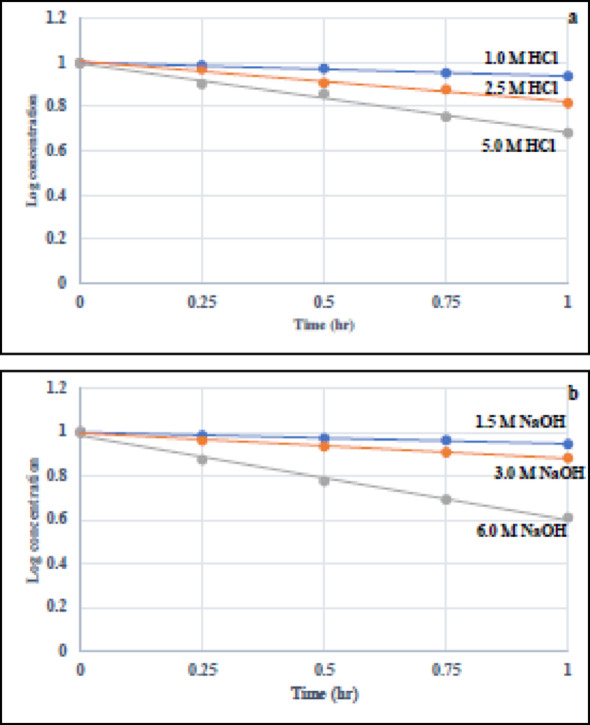
Kinetic study of the acidic (**a**) and alkaline (**b**) hydrolysis of TH at different strengths of HCl and NaOH, respectively

**Fig. 5:**
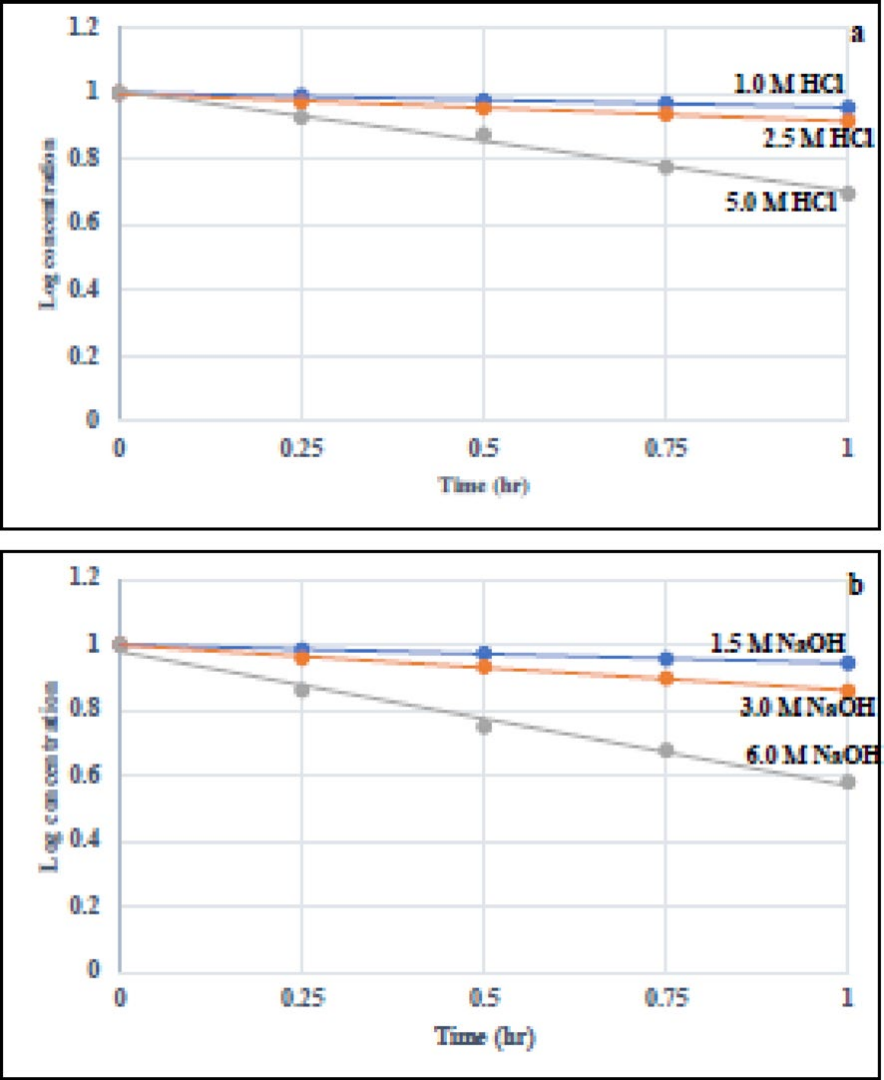
Kinetic study of the acidic (**a**) and alkaline (**b**) hydrolysis of BO at different strengths of HCl and NaOH, respectively

Acidic and basic hydrolysis using different strengths of HCl and NaOH solutions were performed at room temperature for 1 h. Our proposed method was utilized to study the degradation kinetics of both TH and BO. Acidic and basic degradation kinetics were accomplished by computing the remaining concentration of either TH or BO (C_t_) after specified time (t) intervals. Linear relationships were perfectly found by plotting the log remaining concentrations of each analyte versus time using different concentrations of HCl or NaOH (Figs. 4 and 5). The degradation processes were found to follow pseudo first-order kinetics. We could determine the relevant degradation rate constant and half-life (t_1/2_) for each degradation condition, from the slopes of the straight lines, by using the following equations [57, 58]:

$$\log {{\text{C}}_{\text{t}}}=\log {{\text{C}}_0} - {\text{kt}}/2.303$$

$${{\text{t}}_{1/2}}={\text{ }}0.693/{\text{k}}$$

where Ct and C0 are cited drug concentrations estimated at a given time t and at zero time, respectively, k is the apparent first order rate constant and -K/2.303 is the slope of the line.

Table 1 summerizes the rate constants and half-lives under different degradation conditions. It is clearly expected that any increase in the strengths of HCl or NaOH resulted in decreasing the half-lives at room temperature. Both TH and BO were proved to have nearly the same stability under different acidic and basic media as illustrated form their half-lives.

**Table 1:** Degradation results, Pseudo first-order rate constants (K) and half-lives (t_1/2_) for the acidic and basic hydrolysis of TH and BO

Acidic hydrolysis at room temperature						
HCl strength (M)	TH			BO		
	%Remaining after 1 h ± SD	K (h^−1^)	t_1/2_ (h)	%Remaining after 1 h ± SD	K (h^−1^)	t_1/2_ (h)
1.0	87.12 ± 1.57	0.1429	4.85	90.50 ± 1.74	0.1021	6.79
2.5	66.69 ± 1.23	0.4222	1.64	82.12 ± 1.42	0.1915	3.62
5.0	40.19 ± 0.82	0.8864	0.78	49.39 ± 0.98	0.7037	0.98

Basic hydrolysis at room temperature						
NaOH strength (M)	TH			BO		
	%Remaining after 1 h ± SD	K (h^−1^)	t_1/2_ (h)	%Remaining after 1 h ± SD	K (h^−1^)	t_1/2_ (h)
1.5	88.41 ± 1.68	0.1211	5.72	87.85 ± 1.62	0.1292	5.36
3.0	75.68 ± 1.40	0.2675	2.59	72.51 ± 1.35	0.3184	2.18
6.0	39.63 ± 0.72	0.8863	0.78	37.99 ± 0.87	0.9430	0.73

### Method validation

The proposed method was validated as per the principles set out by the newly endorsed ICH Q2(R2) on validation of analytical procedures [59].

#### Linearity

The linearity of the proposed HPTLC procedure was assessed by analysis of a series of serial concentrations of each drug. The peak areas measured were found to be proportional to the drug concentrations. Table 2 illustrates the linearity data and statistical parameters of the proposed method. Good linearity was proved by regression analysis as represented by the correlation coefficient values (*r* ≥ 0.9997) in addition to values of RSD% of the slope which were found less than 0.9%. Figure S5 (in supplementary file 1) illustrates the calibration plots of peak areas versus the corresponding concentrations of the cited drugs.

**Table 2:** Analytical parameters for the determination of TH and BO using the proposed method

Parameters	TH	BO
Range (µg/band)	2.5–30	2.5–20
Intercept (a)	− 43.82	− 7.67
Slope (b)	233.744	262.23
Correlation coefficient (r)	0.99976	0.99986
S_a_^i^	40.71	25.91
S_b_^ii^	2.586	2.226
RSD% of the slope	0.02	0.85
S_y/x_^iii^	60.64	32.45
LOD	0.58	0.33
LOQ	1.74	0.99

^i^Standard deviation of the intercept

^ii^Standard deviation of the slope

^iii^Standard deviation of residuals

#### Limits of detection and quantification

The limits of detection (LOD) and quantification (LOQ) were calculated using 3.3 σ/s and 10 σ/s formulas, respectively, where σ is the standard deviation of the intercept of the regression line and s is the sensitivity (the slope of the calibration curve) [59]. The high sensitivity and sufficient accuracy of the developed method were proved by the values of LOD and LOQ shown in Table 2.

#### Accuracy and precision

The within-day (intra-day) precision (repeatability) of the proposed method was studied at three concentration levels for each compound (each in triplicate determination) through the same day. While, the between day (inter-day) (intermediate) precision was tested by analyzing the same three concentrations (each in triplicate determination) repeated in 3 days [59]. The high precision was proved by the small values of percentage relative standard deviation (RSD %) not exceeding 0.50% (Table S3 in supplementary file 1). Moreover, good accuracy of the proposed method was indicated by the satisfactory recoveries (%R) (99.72%–100.56%) along with its small standard deviation (SD) (less than 0.50) together with the small values of percentage relative error (E_r_ %) (not exceeding 0.60%) (Table S3 in supplementary file 1).

#### Specificity

Method specificity was verified by the determination the intact drugs in the presence of their forced degradation products (Fig. 3). Peak purity of remaining intact drugs [60] was ascertained by comparing the spectra of the sample and standard bands at peak start (s), peak apex (m) and peak end (e) positions on the spot by means of the HPTLC scanner (Figure S6 in supplementary file 1). A good correlation (r (s, m) and r (m, e) not less than 0.9997) was obtained between the standard and the sample spectra of each drug, indicating the absence of any co-eluted degradation products with the intact drug remained after stress degradation (Table S2 in supplementary file 1). Thus, confirmation of purity and homogeneity of the drug peaks by means of HPTLC scanner attested the stability-indicating capability and high specificity of the designed method.

#### Robustness

Robustness of the developed method was studied by making slight deliberate changes in different analysis parameters [59]. Tested parameters were: chloroform volume in the mobile phase (± 1 mL), duration for chamber saturation (± 5 min) and time spent between spotting and development (± 5 min). Table S4 (in supplementary file 1) depicts all results, where small values of RSD% (less than 2) proved the high robustness of the method since small deliberate changes in the analysis parameters did not significantly affect the developed method.

#### Stability of solutions

The stability of working solutions in methanol was verified and found to be stable at room temperature for up to 10 h with no chromatographic changes in the peak areas or R_f_ values. In addition, standard solutions were stable for up to 1 week in case of BO while only for 3 days in case of TH when kept refrigerated at 4 °C. Concerning the eomployed solvents, it is recommended to store methanol and chloroform solvents as well as ammonia solution in tightly sealed containers under appropriate conditions (cool and dark environments) to garanttee their long term stability.

### Applications of the developed method

#### Analysis of laboratory-prepared mixtures

Several laboratory prepared binary mixtures at various ratios of TH and BO with concentrations within the linearity ranges illustrated in Table 2 were assayed according to the previously described procedure. Excellent values of recovered concentrations, RSD % and Er % proved the precision and accuracy of the developed method and illustrated their ability to resolve and quantify different mixtures of the cited drugs in various ratios Table S5 (in supplementary file 1). Furthermore, representative densitograms of the synthetic mixtures of TH and BO prepared in the same ratio found in the combined marketed capsules (TH: BO, 100:1, w/w) are illustrated in Figure S7 (in supplementary file 1).

#### Assay of fresh Thioglu^®^ capsules

The developed method was applied for the assay of the cited drugs in their valid commercial pharmaceutical formulation (Thioglu^®^ capsules). TH and BO corresponding peaks eluted at their specific R_f_ values with no observed interfering peaks from any of the excipients that may be present in the preparation (Figure S8 See Supplementary File 1). Recoveries calculated using both external standard and standard addition methods were gathered in Tables S6 and S7 (in supplementary file 1). The assay results were found precise, accurate and in agreement with the label claim.

Analogously, peak purity for TH and BO in the assayed Thioglu^®^ capsules extract samples was performed by comparing the R_f_ and spectra of both drugs peaks in the analyzed capsules extracts with those of the standard samples of both drugs where good correlations (r (s, m) and r (m, e) not less than 0.99982) were obtained indicating no interference or co-elution of any of the existing excipients with the analyzed drugs (Figure S9 in Supplementary File 1).

#### Analysis of expired Thioglu^®^ capsules

The specificity of the proposed method was further demonstrated through the analysis of expired Thioglu^®^ capsules (≈ 3 years after expiry) stored at ambient temperature under normal conditions. Figure S10 (in Supplementary File 1) presents the densitograms of pure and expired stability samples of TH and BO where about 45 ± 2% and 40 ± 1.7% of TH and BO, respectively, were recovered with complete separation of the cited drugs from all existed degradation products. Moreover, confirmation of purity and homogeneity of the drug peaks by means of HPTLC scanner was carried out attesting the stability-indicating capability and high specificity of the proposed method (Figure S11 in Supplementary File 1). It is noteworthy to mention that two of the degradation products’ peaks of expired samples appeared at the same retention factor of hydrolytic degradation products’ peaks of both drugs, this encourages further investigation of the chemical structure of these generated degradation products in aged capsules in future work. This represents an interesting illustration of the utility of stress degradation study in providing an approximate reflection of actual drug degradation under shelf conditions.

Regarding the analysis of expired finished pharmaceutical products (FPPs), similar studies were previously reported along the last few years [61–65].The aim of this type of studies is to check the stability and to determine the content of active pharmaceutical ingredient (API) in the FPPs beyond their expiration dates. Interestingly, the results of many of these performed studies revealed that most of the APIs retain their pharmacological potency far beyond their claimed expiration date including many studies conducted by the FDA [63–65].The current performed study represents just a pilot study to verify the ability of the designed HPTLC method to be implemented in the analysis of combined Thioglu^®^ capsules even beyond their expiry date also for post-market surveillance and regulatory verification. Noteworthy, similar studies found in the scientific database employed HPLC-DAD, HPLC-MS or HILIC connected with MS, hence the herein proposed method represents the first developed HPTLC method for testing expired FPPs and determining its APIs content with neat separation of the peaks of degradation products generated during aging of the capsules. Indeed, the HPTLC technique possesses several merits over the HPLC-DAD and HPLC-MS techniques specially from economic point of view as previously discussed under “Results and discussion” and these merits emphasizes the real world cost benefits of future implemention of the proposed method at a larger scale.

### Tri-faceted evaluation and comparison of sustainability of proposed method with published methods

The progressive tremendous industrial development has contributed to huge negative environmental impact including ecological pollution and horrific climate changes. This encouraged a global rise in interest in green analytical chemistry (GAC) [66] and the more insightful and comprehensive alternative; white analytical chemistry (WAC) [48] as an attempt to reduce the damage caused to the environment. Lately, the newly designed analytical methods should be evaluated as per their conformity to the 12 principles of GAC and the more meticulous 12 tenets of WAC. This help in the selection of the optimal method for the analysis of the analytes of interest with best compromise between analytical performance, eco-compatibility and aptness for a given application. The herein performed tri-faceted evaluation assesses and compares the method’s greenness (eco-compatibility), blueness (functionality) and whiteness (sustainability) with those of the reported HPLC qualitative methods [37, 38] because, So far, no quantitative analytical methods for simultaneous determination of both drugs were published.

#### Evaluation of the method’s greenness and comparison with published methods

To assert that the developed method is green, several complementary greenness metrics should be applied so that the coherence and synergy of their good results provide a pragmatic evidence on its good green character.

The most commonly applied Analytical Eco-Scale [44], AGREE [45, 67] and the recently endorsed MoGAPI (September 2024) [46] metrics are applied to assess and compare the method’s greenness.

The Analytical Eco-Scale represents a mathematic calculation assuming that the ideal green analysis possesses a score of 100. This tool assigns penalty points to each character that departs the method from being ideal green. These characters include the hazards of employed reagents and solvents, threats affecting the operator, volume of waste output and energy consumed by the applied technique. Then finally these penalty points are deducted from 100 to generate the eco-scale score. Methods obtaining scores above 75 are regarded as excellent green methods. Adequate green character is assigned to methods that acquired scores between 75 and 50, while those with scores less than 50 are regarded as inadequate green methods. After application of this tool, the proposed HPTLC method obtained a score of 80. The reported ion interaction based HPLC method [38] acquired a score of 86 and the avidin binding based HPLC method [37] obtained a score of 83, thus the 3 methods are regarded as excellent green (Table 3 and S8 (in supplementary file 1)).

**Table 3:**
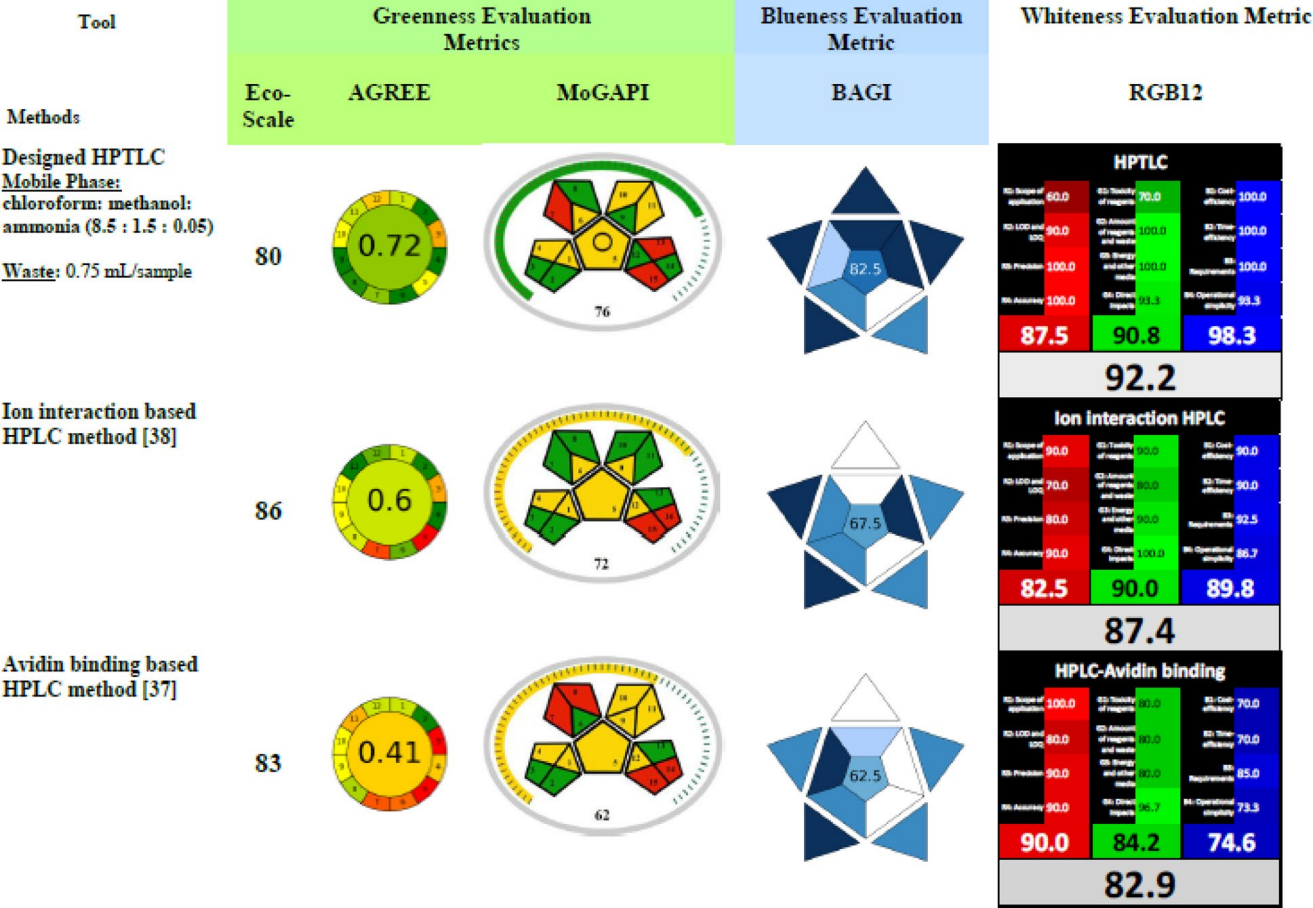
Tri-faceted color evaluation of sustainability of designed HPTLC method and comparison with reported HPLC methods [37, 38]

AGREE represents a more comprehensive and informative tool. It assesses 12 parameters of the analytical method related to the 12 GAC principles and includes many disregarded parameters by the eco-scale tool. The 12 AGREE assessed parameters include the amount of analyzed sample, the positioning of the device and its automation degree, degree of integration of analytical process steps, employment of derivatization step, volume of analytical waste, analytical throughput and operational safety. The result is then straightforward generated in the form of circular pictogram possessing a final central score (from 0 to 1) and surrounded with 12 colored segments (green or yellow or red) relevant to the 12 assessed parameters. As the final score is near to 1, the greener is the method. Upon application of this metric, the proposed method acquired the highest green score (0.72). While, the reported ion interaction based HPLC method [38] acquired a less green score of 0.60 and the avidin binding based HPLC method [37] obtained the lowest score of 0.41 denoting its inadequate green character (Table 3) (all input data of AGREE metric are represented in supplementary file 2).

Finally, the most recent MoGAPI metric was applied. This tool is a modified more advanced alternative to the conventional GAPI tool [68]. It resembles the conventional GAPI in assessing 15 ecologically effective characters of the analytical procedure. It assesses the entire methodology starting from the sampling step going through the transport, storage, sample preparation steps, health and safety hazards of employed reagents till reaching the final determination step and volume of waste. The MoGAPI excels over the conventional tool in the following merits: it employs user-friendly software which calculates a final numeric score and generates the five colored pentagrams encompassing the 15 colored zones (green, yellow or red) relevant to the 15 evaluated attributes. It also provides a holistic colored scale (green, yellow or red) surrounding the pentagrams and affords an overall visual evaluation of the procedure. Numeric scores ≥ 75 with green overall scale indicate excellent green character, scores from 50 to 74 and yellow general scale denote acceptable green method, while scores less than 50 with red holistic scale declare inadequate green method. Following the application of this advanced insightful tool, akin to previous results, the proposed method acquired the highest score of 76 with green holistic scale. The reported ion interaction based HPLC method [38] acquired a score of 72 with yellow holistic scale and the avidin binding based HPLC method [37] obtained lowest score (62) with yellow holistic scale (Table 3) (all input data of MoGAPI metric are represented in supplementary file 2).

These results were attributed to the following facts: although the proposed HPTLC method employs hazardous solvents (chloroform, methanol and ammonia), this does not greatly affected its green character because a very small volume is needed for the analysis of each sample because of the high analytical throughput of the method (40 samples are analyzed per hour). Consequently, minute volume of waste is generated per sample, since only 15 mL of mobile phase are needed for the analysis of 20 samples (0.75 mL waste/sample). The aforementioned merits are generated from the ability of the HPTLC technique to concurrently analyze up to 20 samples on each single TLC plate in a parallel way in very short time consuming the lowest energy and generating the lowest waste volume when compared to the reported HPLC techniques [37, 38]. The energy consumed by HPTLC is significantly lower than that consumed by HPLC technique. HPTLC uses only the UV scanner for the detection of separated peaks and quantification, which is operated for only few seconds at the end of the experiment and less than 5 min are needed for operation of the automatic syringe during spotting 20 samples on the TLC plate (automated syringe spotting rate is 1.2 µL/second). Additionally, HPTLC does not use a quaternary high energy pump, degasser system and loop injector system which all operate during preconditioning time and the whole HPLC chromatographic procedure as well as between chromatographic runs.

#### Evaluation of the method’s blueness and comparison with published methods

BAGI [47] represents a recently established user friendly metric that provides a quantitative and qualitative evaluation of the analytical method’s practicality. It focuses on depicting how practical and fit-for-purpose is the methodology in real world application by appraising 10 attributes related to sample preparation and analytical determination steps. This tool scrutinizes the type of analytical technique, the number of concurrently estimated analytes, the instrumentation needed, the type of employed reagents and their availability, sample amount, degree of automation of applied technique and analytical throughput of the method. After feeding the software with the needed data, an asteroid pictogram is automatically generated consisting of 10 colored parts from dark blue, blue, light blue to white denoting excellent, medium, low or no compliance to the functionality aspects, respectively. Moreover, a numerical BAGI score (ranging from 25 to 100) is provided in the center of the pictogram to afford a quantitative description of the degree of practicality of the method. The methodology is regarded practical when it attains a final BAGI score greater than 60.

After evaluating the three methods using the BAGI tool, the proposed HPTLC method attained a score of 82.5 denoting that it perfectly fits the planned application and possesses excellent practicality in real world application. In contrast, the reported ion interaction based HPLC method [38] obtained a score of 67.5 and the avidin binding based HPLC method [37] acquired a score of 62.5 which highlights their poor functionality and reduced feasibility to the planned application in quality control labs (Table 3) (all input data of BAGI metric are represented in supplementary file 2).

It is worth emphasizing that the aforementioned results are attributed to the following merits of the proposed HPTLC method including: high speed of analysis, both quantitative and confirmatory property, high analytical throughput (40 samples are analyzed per hour), simple instrumentation and commercial availability of the employed reagents and solvents. On the other side, both reported HPLC methods suffer from the following disadvantages including: they are only qualitative methods, they employ greater sample volumes and generate greater waste volume. In addition, one of the reported HPLC methods [37] requires binding of the analytes to avidin prior to detection which renders the analysis more rigorous and hence it obtained the lowest BAGI and greenness scores (Table 3).

#### Assessment of whiteness of the proposed method and comparison with reported ones

White Analytical Chemistry (WAC) [48] symbolizes a more holistic approach which reconciles between 3 complementary pillars decoding the analytical effectiveness, eco-compatibility and practicality features needed to assign a sustainable character to analytical method. WAC assesses 12 features of the analytical method including 4 inclusive red characters symbolizing the analytical efficacy of the method, 4 overarching green attributes appraising the method’s eco-compatibility and 4 blue aspects judging its functional and economic features. This is totally expressed as the RGB 12 algorithm. All data are fed in an excel spread sheet and results are generated as three separate scores (ranging from 0 to 100) for each assessed pillar (red, green and blue) along with an overall mean score (ranging from 0 to 100) denoting the method’s final whiteness score. After performing this comprehensive evaluation, the proposed method acquired the highest whiteness score (92.2) followed by the reported ion interaction based HPLC method [38] (87.4) and, akin to previous results, the avidin binding based HPLC method [37] got the lowest score (82.9) (Table 3, S9 and S10 (in supplementary file 1)).

Undoubtedly, the previous results emphasize the proposed method’s excellent analytical reliability, good eco-compatibility and great economic feasibility. It can be described as a well-balanced tri-faceted methodology fitted for its planned application in quality control labs.

## Future work recommendations

Although the presented study demonstrates excellent performance in resolving the stress degradation products of TH and BO which reflects the marked selectivity of the designed method, other approaches are still remaining for future work enhancement. Particularly, further chemical separation and structural elucidation of the formed degradation products are recommended using structural elucidative technique such as MS (mass spectrometry) and IR (infra-red spectrometry). This could help in establishing the possible degradation pathways of the studied drugs under various hydrolytic conditions. Moreover, testing the stability of the cited drugs under other stress degradation conditions such as dry heat (oven) and oxidation are necessary to be implemented in future work to provide a thorough stability study of the cited drugs. Also, investigating the photostability of the tested analytes (under sunlight and/or UV exposure) would enrich the scientific value and practical relevance of the study, especially in terms of formulation development and storage recommendations. By addressing these expanded studies, the designed method could then tolerate a more difficult quality control challenges concerning the selective quantification of impurities in pharmaceutical manufacturing.

Future work could be greatly improved through application of the Analytical Quality by Design (AQbD) approach [69]. AQbD uses risk management and experimental design to develop more robust and reliable methods, aligned with ICH Q14 guidelines. It also enhances method understanding, reduces the need for revalidation, and ensures long-term method suitability. In future studies, we are planning to adopt this strategy for better analytical outcomes.

Additionally, the performed analysis of expired combined capsules provides just an interesting pilot study. It affords documented evidence on the ability of the designed method to selectively determine the remained intact portion of the studied drugs even in presence of the formed degradation products upon aging of the capsules beyond their expiry date. Further extended studies are essential to verify additional characteristics of the FPPs other than the content of APIs such as physical characteristics (content uniformity, sample homogeneity, release rate and dissolution studies). Also, microbiological stability and clinical efficiency of expired capsules could be tested. Such extended studies support broader application in quality control and post-market surveillance. Moreover, risk assessment regarding the implications of using expired drug formulations in clinical settings is required. This includes considerations of chemical stability, efficacy, safety, regulatory concerns and potential toxicity of degradation products even if existing in minute amount.

Based on the results obtained in the current study, it is preferred to conduct accelerated stability studies on the FPPs and longer stability study on freshly expired FPPs which are expected to maintain their potency for several months beyond their claimed expiry date. Such results may encourage regulatory authorities to reconsider and expand the shelf life of this formulation. Generally, the expansion of expiration dates of outdated FPPs was previously reported for other medications [70, 71] and would be economically very beneficial for health care systems, pharmacists and stockpile managers specially in developing countries, since the rate of replacement of stockpiled drugs could be prolonged. Nevertheless, some challenges may be confronted upon scaling up this approach for commercial or industrial applications. These include consideration of the potential cost implications of raw materials and processing steps, and regulatory compliance requirements. Additionally, challenges such as equipment limitations in quality control units existing in manufacturing companies, and waste management.

## Conclusion

The present HPTLC method was proved to be sensitive, accurate and able to discriminate between TH, BO and their different hydrolytic degradation products. Scientific database did not reveal any analytical report attempting the stability testing and simultaneous determination of both drugs. Forced degradation studies on both drugs revealed that wet heat, acidic and alkaline hydrolysis cause variable degradations, with a loss of ~ 4 to 20% of the drugs’ potencies. The method can be applied to determine the purity of TH and BO available from various suppliers. The high specificity of the proposed method was illustrated through the neat separation together with accurate quantitation of the cited drugs in presence of their degradation products existed either in stress degraded or aged stability samples. Furthermore, after performing a thorough, unbiased, multi-metric tri-faceted and complementary assessment of sustainability, the herein proposed method proved to be a well-balanced green, blue and white methodology. In other words, the proposed method is deemed as a well fitted method for its planned application and hence feasible for implementation for routine analysis and stability testing of the cited drugs in quality control labs. Moreover, the acidic and alkaline degradation kinetics were studied to calculate the degradation rate constants of the studied drugs as well as their half-lives. It is worth noting that, the proposed method is regarded as the first report tackling TH and BO degradation kinetics.

## Supplementary Information

Supplementary Material 1.

Supplementary Material 2.

## Data Availability

All data generated or analyzed during this study are included in the published article and supplementary information files.
