# Supplementary material for: Hydrolytic stress degradation study and concomitant HPTLC estimation of thioctic acid and biotin in their combined capsules: greenness, blueness and whiteness assessment
Source: BMC Chem. 2025 Oct 29;19(1):290. doi: 10.1186/s13065-025-01637-5 (PMC12574119; doi:10.1186/s13065-025-01637-5)
Supplement: Supplementary file 1 — Supplementary Material 1. [file 13065_2025_1637_MOESM1_ESM.pdf]

Supplementary File 1

Supplementary Figures

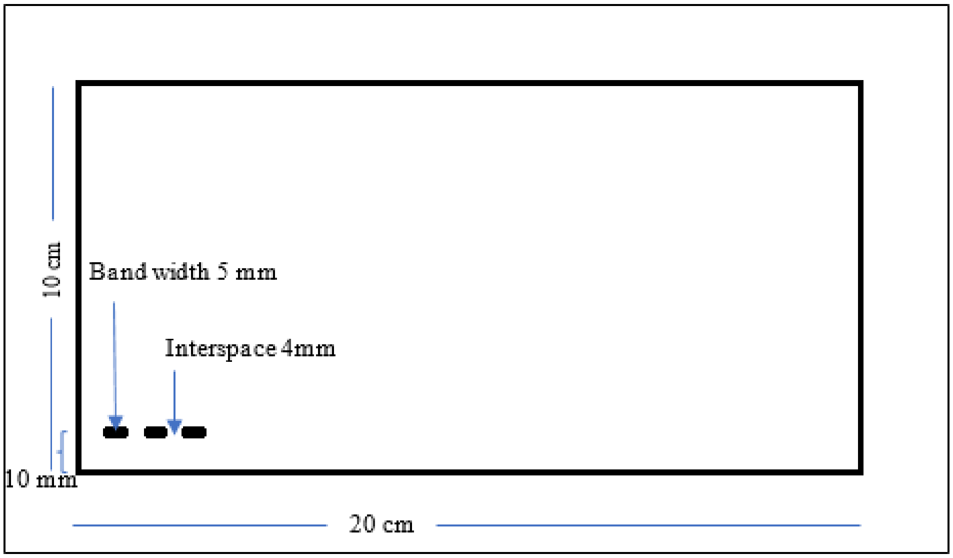

Figure S1: diagram of all the characteristic features of spotting procedure and band separation on the TLC plate.

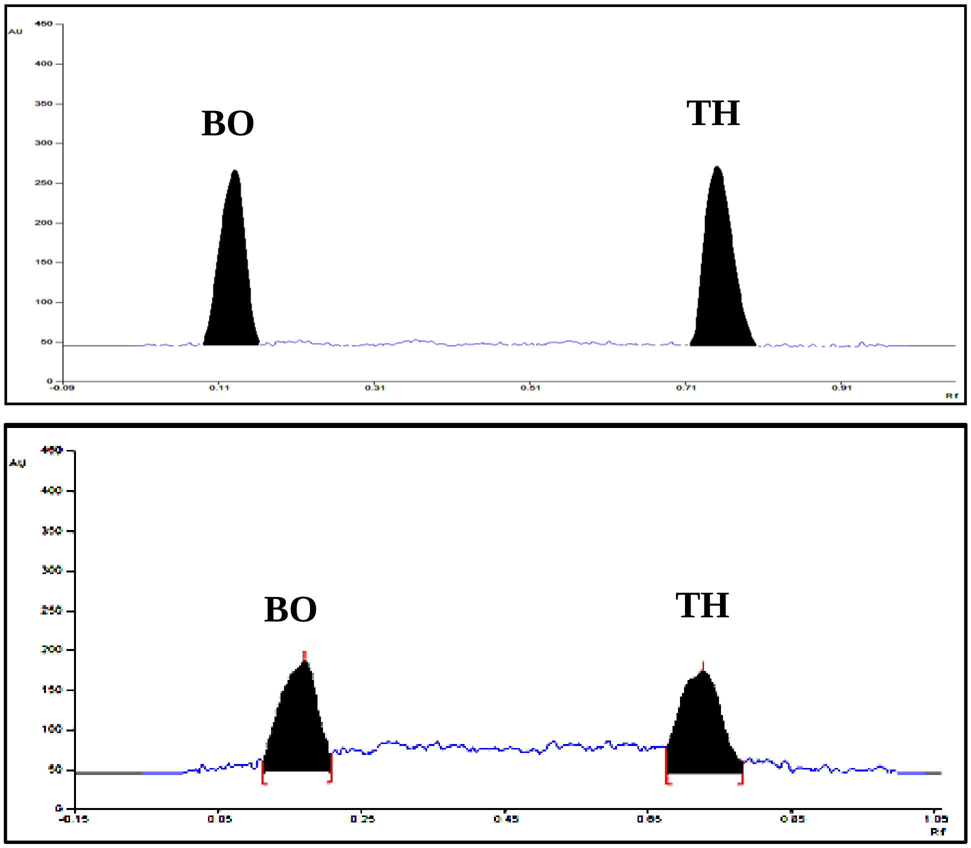

Figure S2: Densitograms of pure mixture of TH (20μg/band) and BO (20μg/band) using activated TLC plates (a) and non-activated plates (b).

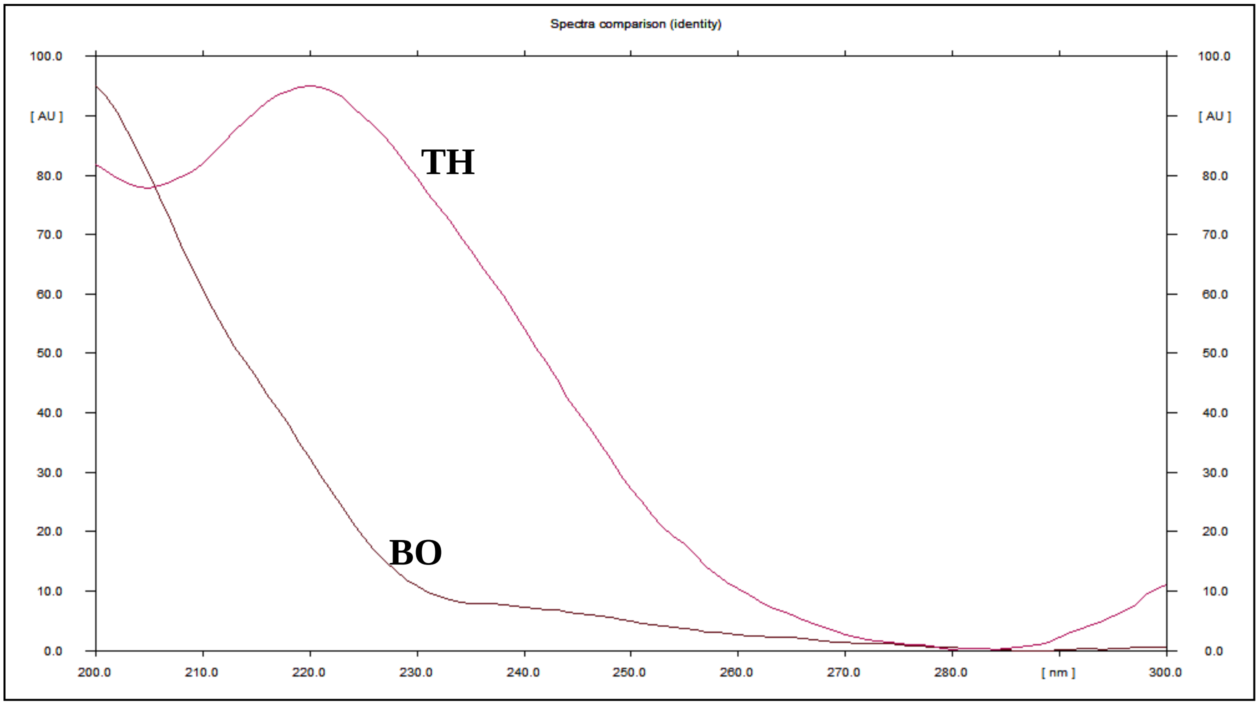

Figure S3: UV spectra of TH 1.5 mg/mL and BO 1.5 mg/mL.

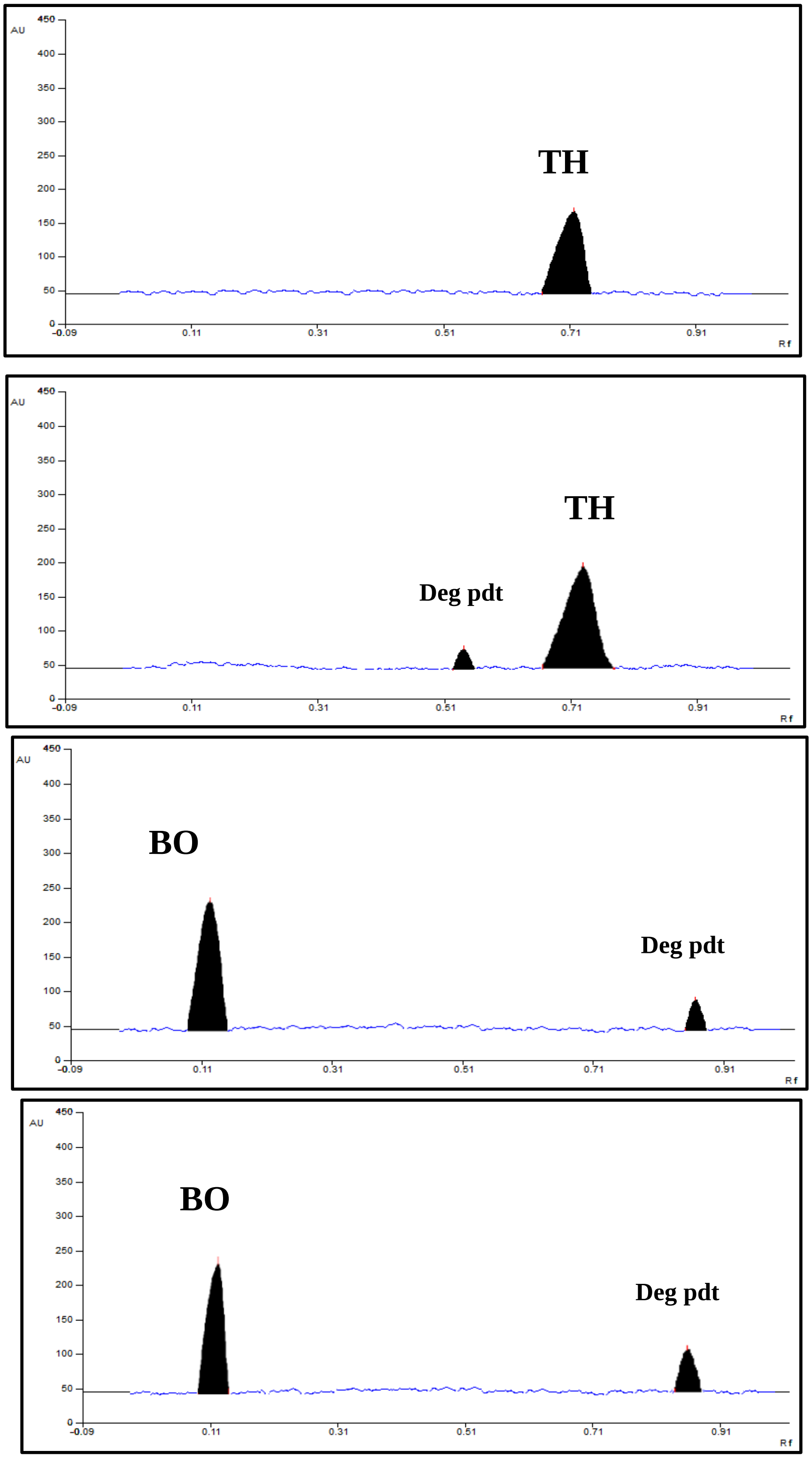

Fig S4: Densitograms for TH (15µg/band) under acidic (a) and basic (b) hydrolysis and for BO (15µg/band) under acidic (c) and basic (d) hydrolysis.

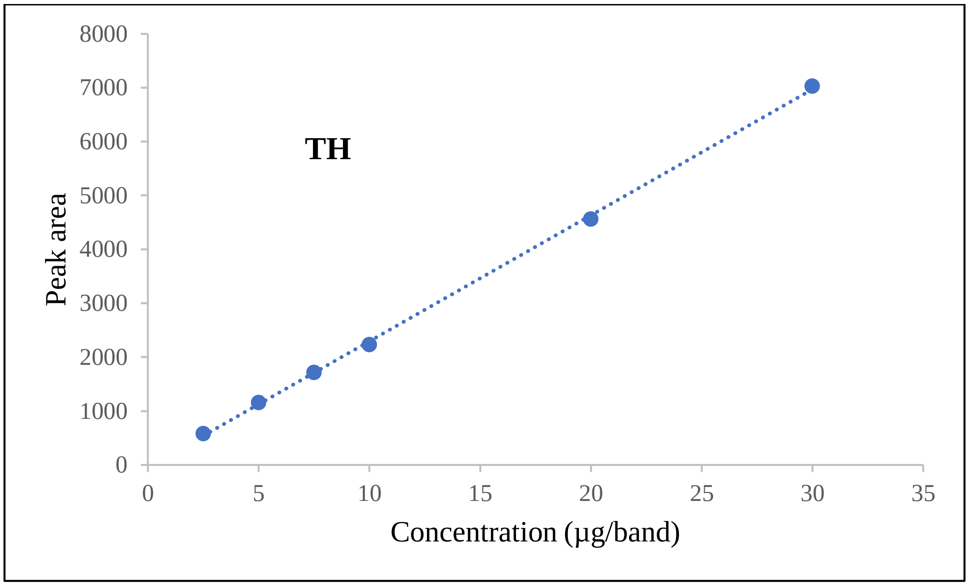

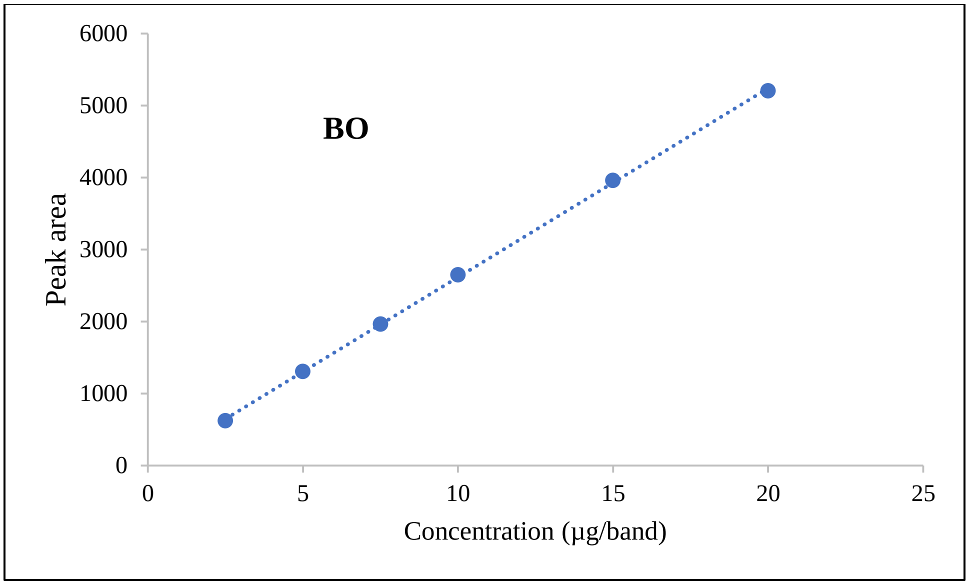

Figure S5: Calibration plots of TH and BO

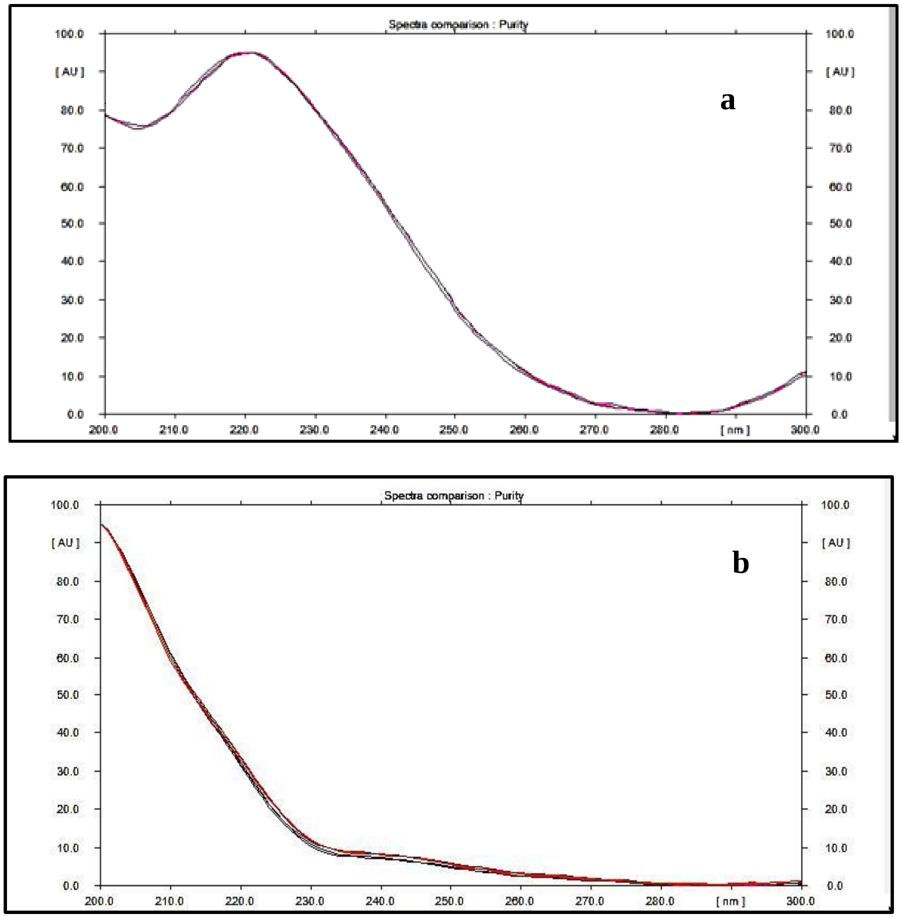

Figure S6: Peak purity profiling by the HPTLC scanner of UV spectra of standard and remained intact TH (a) and BO (b) after various hydrolytic stress conditions.

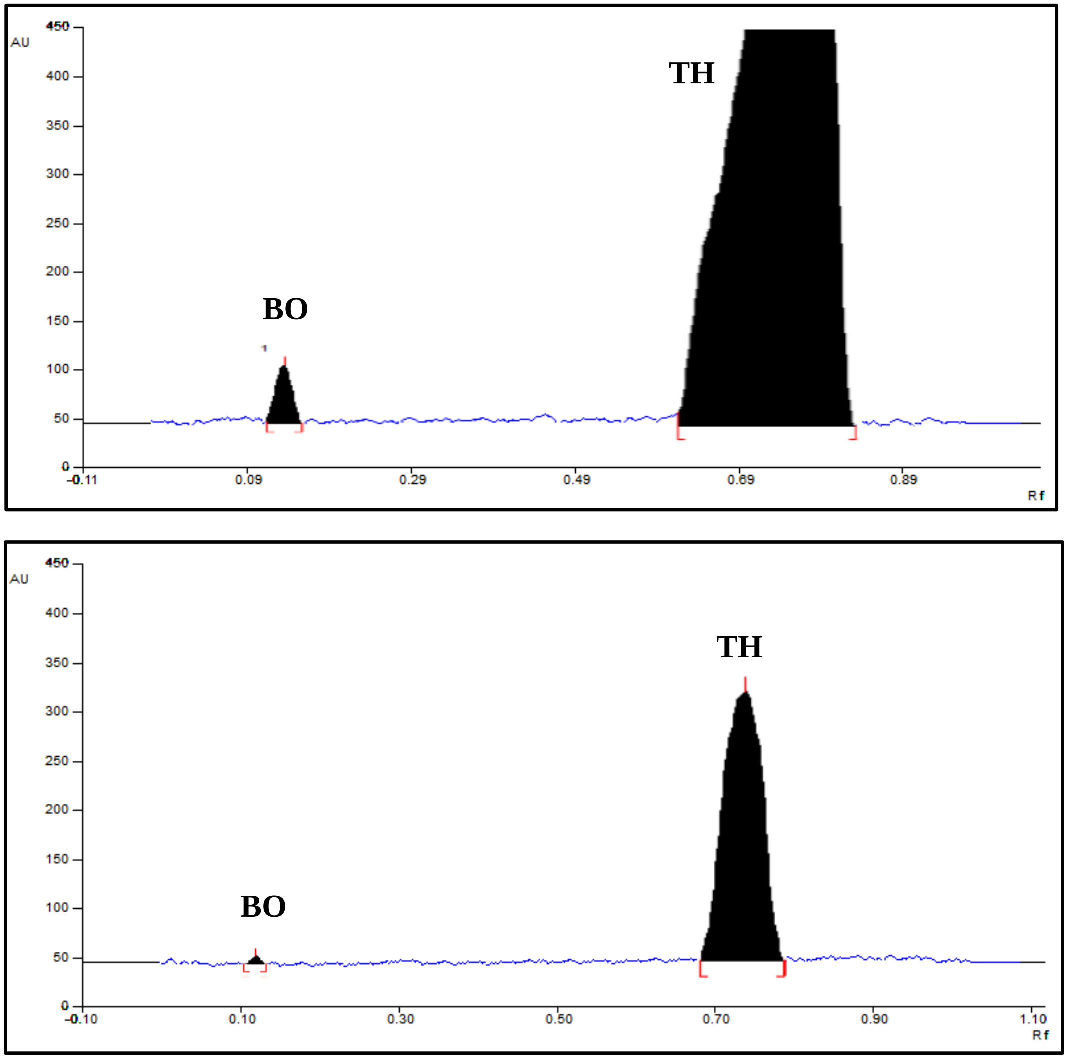

Figure S7: Densitograms of pure mixture of TH:BO, 300:3 µg/band (a) and pure mixture of TH:BO, 30:0.3 µg/band (b).

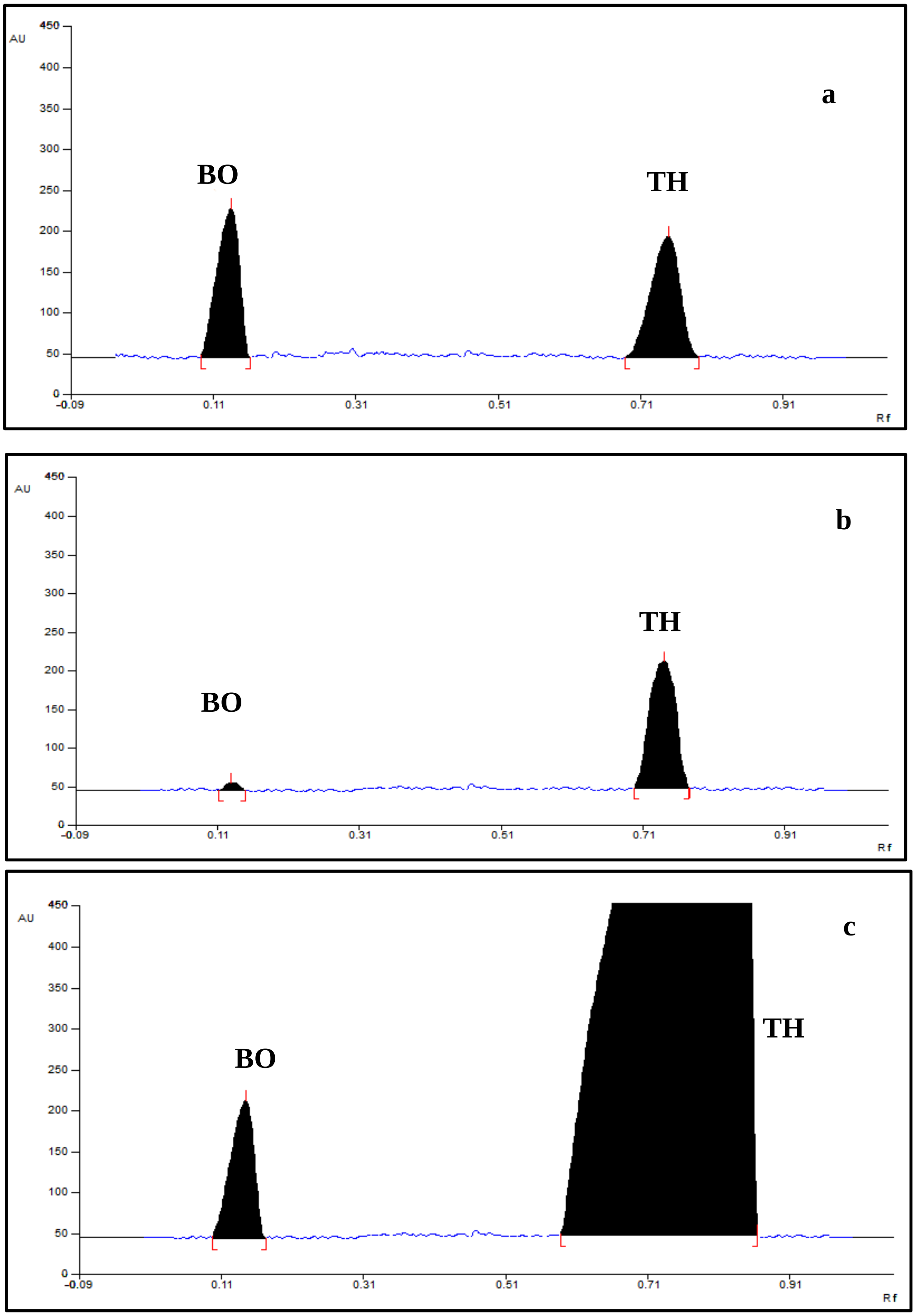

Figure S8: Densitograms of pure mixture of TH (15µg/band) and BO (15µg/band) (a) valid capsule extract for TH determination (b) valid capsule extract for BO determination (c)

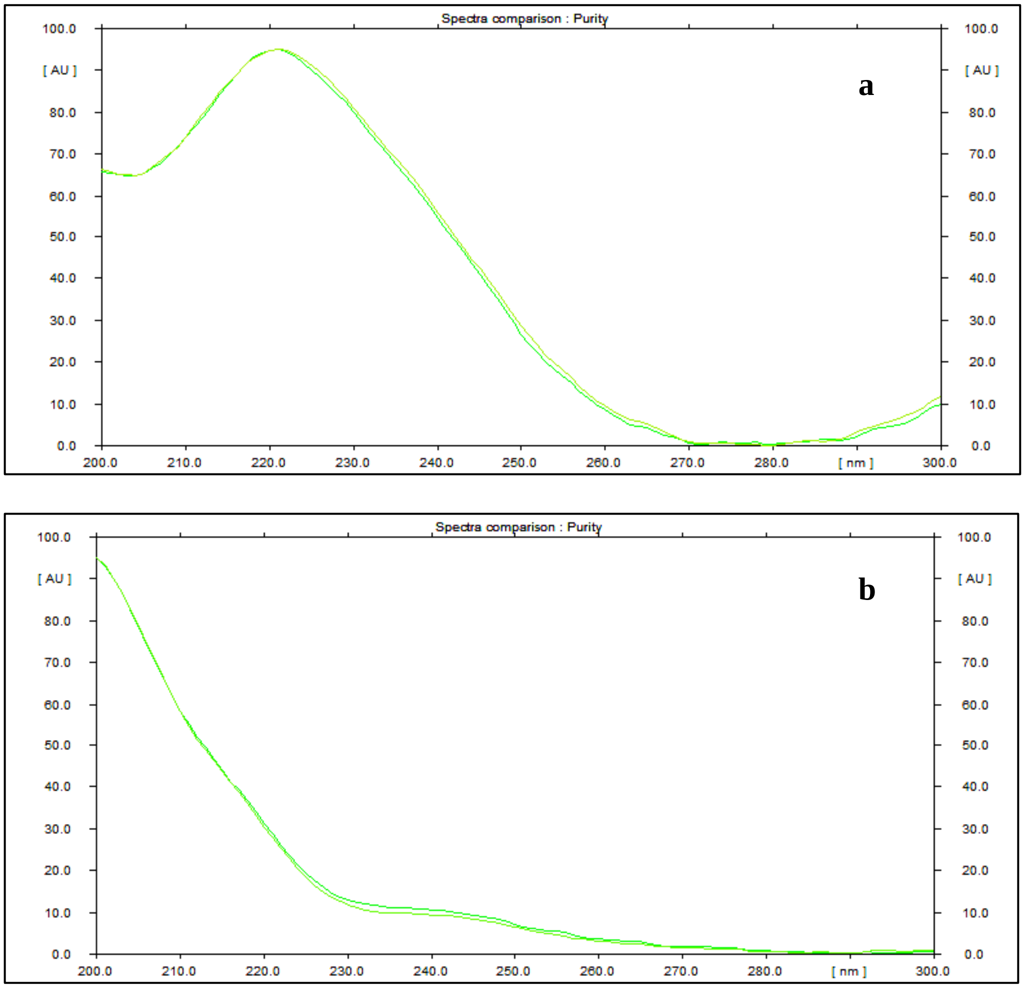

Figure S9: Peak purity profiling by the HPTLC scanner of UV spectra of standard and valid capsule extract solutions of TH (a) and BO (b).

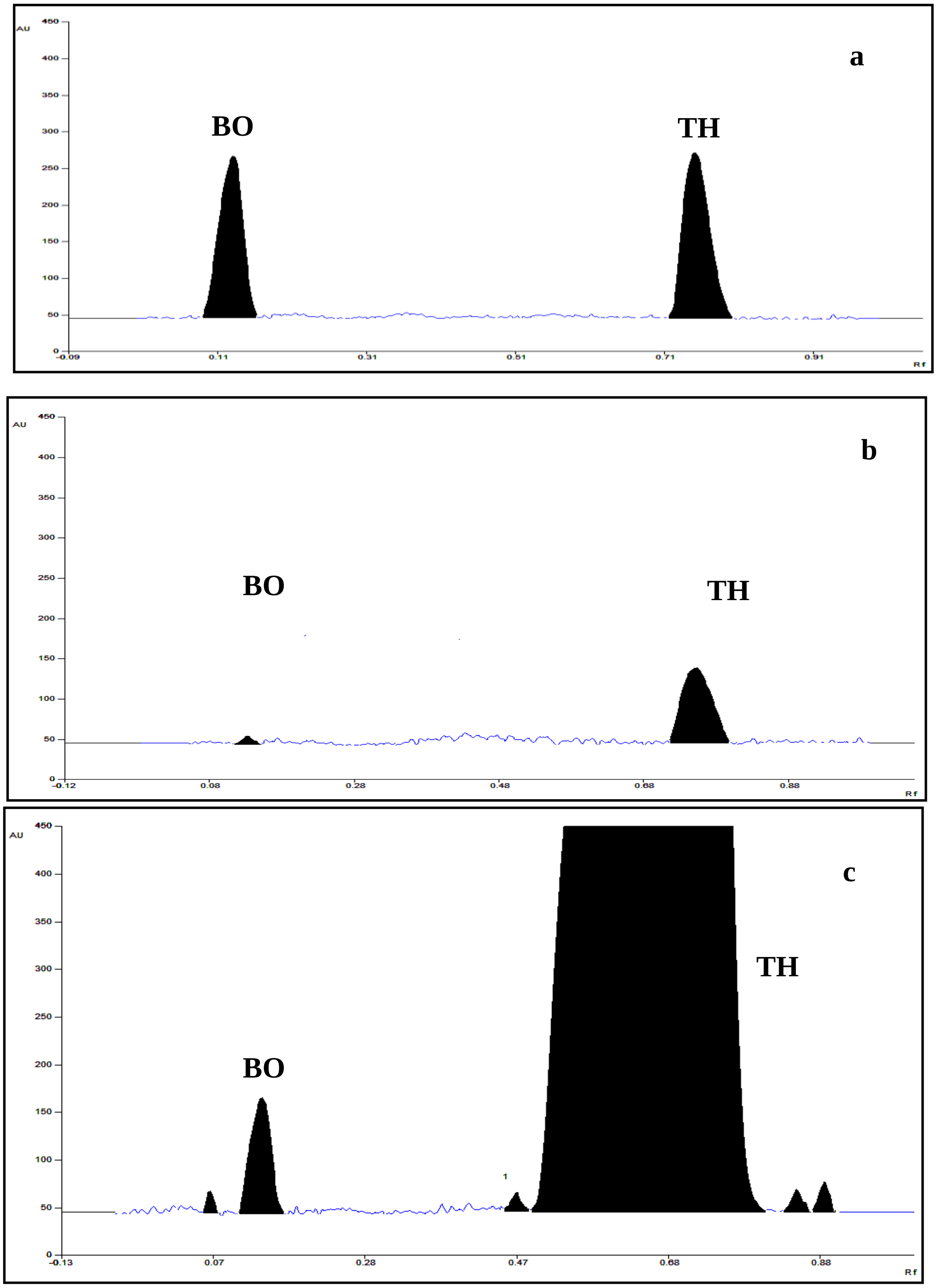

Figure S10: Densitograms of pure mixture of TH (20µg/band) and BO (20µg/band) (a) expired capsule extract for TH determination (b) expired capsule extract for BO determination (c)

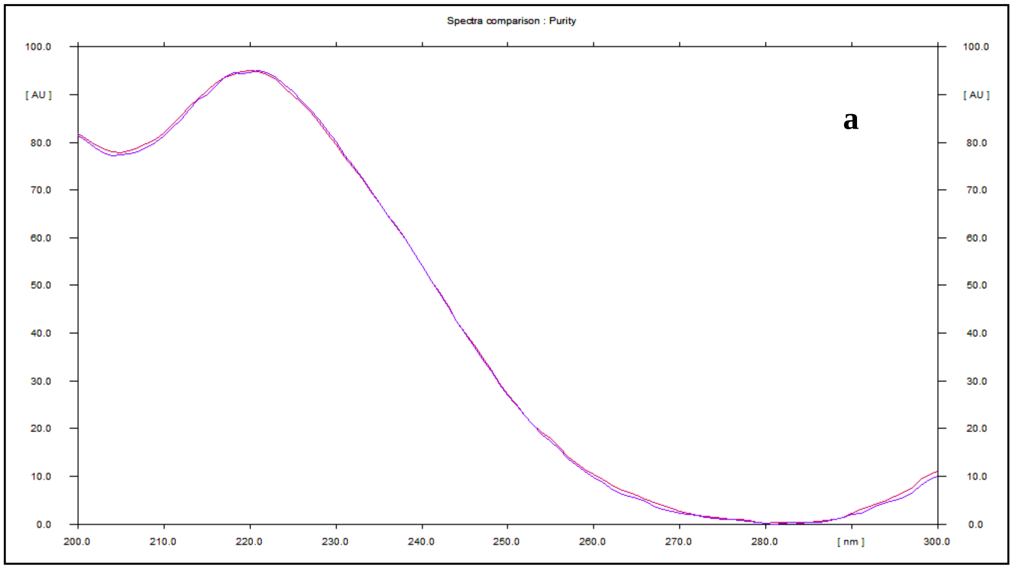

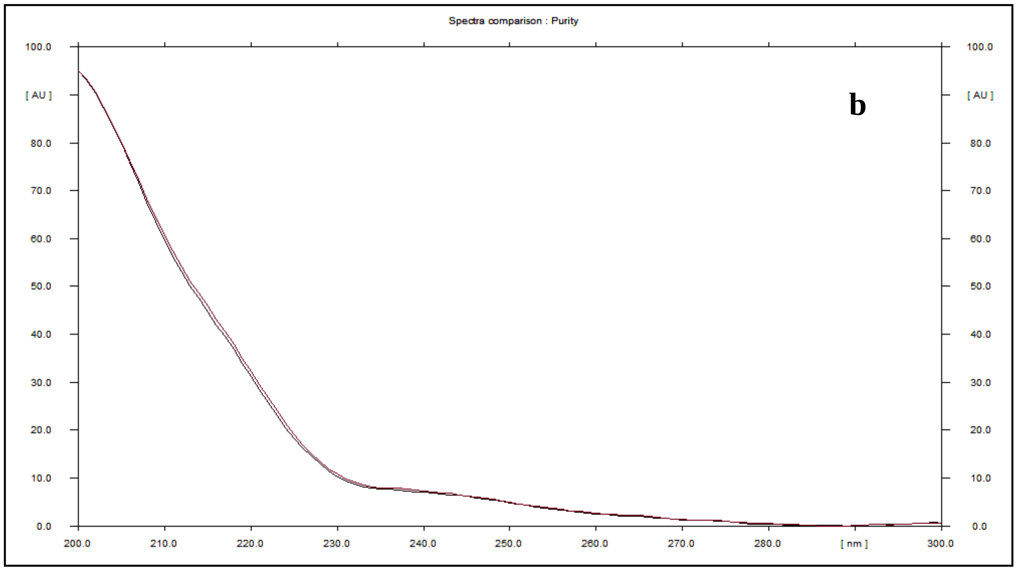

Figure S11: Peak purity profiling by the HPTLC scanner of UV spectra of standard and remained intact drug in expired capsule extract solutions of TH (a) and BO (b).

Supplementary Tables

Table S1: System suitability parameters of the proposed HPTLC method.

Parameter	TH	BO	Reference values [52]
Retention factor (R_f)	0.75 ± 0.02	0.12 ± 0.02	0.1 - 0.9
Symmetry[a]	0.91	0.96	≈ 1
Tailing factor[b]	0.92	1.00	0.9 - 1.1
Capacity factor (k')[c]	0.33	7.33	0.1 - 9
Selectivity (α)[d]	22.21		> 1
Resolution (Rs)[e]	9.86 ± 0.02		> 1

[a]Symmetry factor = b/a, the ratio of back to front width at 10% of peak height.
[b]Tailing factor; T = (a + b)/2 a, at 5% of peak height.
[c]Capacity factor; $k' = (1 - R_f) / R_f$.
[d]Selectivity; $\alpha = k'2 / k'1$.
[e]Resolution; Rs = (Z2-Z1) / 0.5 (W1+W2), where Z is the distance travelled by the analyte from the start line and W is the peak width at 5% of peak height.

Table S2: Summary of the degradation results of TH and BO

Degradation Type	Degradation Conditions	% Remaining[a] ± SD[b]		R_f of degradation peaks		Peak purity index values			
						TH		BO	
		TH	BO	TH	BO	r (s, m)	r (m, e)	r (s, m)	r (m, e)
**Thermal (wet heat)**	100 °C for 5 h	97.40 ± 1.54	96.50 ± 1.47	——	——	0.99991	0.99984	0.99995	0.99982
**Acidic (2.5 M HCl)**	Room temperature for 30 min	81.10 ± 1.26	89.88 ± 1.36	——	0.86	0.99998	0.99997	0.99992	0.99974
**Basic (3.0 M NaOH)**	Room temperature for 30 min	86.03 ± 1.38	85.58 ± 1.22	0.53	0.86	0.99996	0.99998	0.99992	0.99994

[a] Mean of three determinations
[b] SD of three determinations

Table S3: Precision and accuracy for the analysis of TH and BO in their bulk form using the proposed method.

Drug	Conc. (μg/band)	Type of analysis	Mean % recovery	SD	RSD%	E_r%
TH	7.5	Intra-day	100.34	0.16	0.16	0.34
	15		100.12	0.11	0.11	0.12
	25		100.37	0.18	0.17	0.37
	7.5	Inter-day	100.42	0.45	0.45	0.42
	15		100.11	0.48	0.48	0.11
	25		99.80	0.35	0.35	-0.20
BO	5	Intra-day	100.28	0.17	0.17	0.28
	10		100.02	0.09	0.09	0.02
	15		99.72	0.17	0.17	-0.28
	5	Inter-day	99.87	0.30	0.30	-0.13
	10		100.56	0.16	0.16	0.56
	15		100.24	0.37	0.36	0.24

Table S4: Robustness study for the proposed method

Parameters	TH			BO		
	Peak area[a] ± SD[b]	RSD% of peak areas	R_f ± SD	Peak area ± SD	RSD% of peak areas	R_f ± SD
**Chloroform volume** CH_3OH: ammonia: $CHCl_3$ (1.5:0.05:7.5, 1.5:0.05:8.5, 1.5:0.05:9.5)	2251.67 ± 29.02	1.29	0.72 ± 0.01	2641.20 ± 48.73	1.85	0.12 ± 0.002
**Duration of saturation** (15, 20, 25 min)	2232.33 ± 25.50	1.14	0.76 ± 0.02	2656.67 ± 31.57	1.19	0.12 ± 0.002
**Time between spotting and development** (5, 10, 15 min)	2247.33 ± 36.20	1.61	0.77 ± 0.02	2662.00 ± 33.45	1.26	0.12 ± 0.002

[a] Mean of three determinations
[b] SD of three determinations

Table S5: Determination of TH and BO in laboratory-prepared mixtures using the proposed method.

Concentration (µg/band)		TH			BO		
**TH**	**BO**	**Mean[a] %Rec**	**RSD%[b]**	**E_r%**	**Mean %R**	**RSD%**	**E_r%**
5	7.5	100.02	0.40	0.02	99.86	0.21	-0.14
10	10	100.34	0.74	0.34	100.00	0.05	0.00
20	10	100.03	0.46	0.03	100.00	0.14	0.00
7.5	15	99.97	0.50	-0.03	100 .00	0.30	-0.24
30	20	100.38	0.16	0.38	100.04	0.13	0.04
30	0.3	100.21	0.30	0.30			
300	3				100.47	0.18	0.47

[a] Mean of three determinations
[b] RSD% of three determinations

Table S6: Application of the proposed method to the analysis of TH and BO in their combined capsules using external standard method.

Thioglu® capsules	TH	BO
%Recovery	99.89	99.96
SD[a]	0.27	0.33
RSD%	0.27	0.33

[a] Mean ± standard deviation for five determinations
[b] % Relative standard deviation.

Table S7: Application of the proposed method to the analysis of TH and BO in their combined capsules using standard addition method.

Standard added %*	Content (µg/band)	%Recovery ± SD[a]	RSD%[b]	Content (µg/band)	%Recovery ± SD[a]	RSD%[b]
	TH			BO		
**0**	10.00	100.06 ± 0.17	0.17	5.00	100.09 ± 0.18	0.18
**50**	15.00	100.21 ± 0.08	0.08	7.50	100.33 ± 0.15	0.15
**100**	20.00	100.10 ± 0.27	0.27	10.00	100.18 ± 0.36	0.36
**200**	30.00	100.04 ± 0.56	0.56	15.00	100.24 ± 0.33	0.33

*Percentages in the first column represent the quantity of standard added for TH and BO separately during its standard addition procedure. Due to their disparate concentration ratio (100: 1, TH: BO), the dosage form was first extracted to obtain a stock sample solution for BO, which was subsequently employed for the standard addition assay by adding BO standard solution at three levels (50%, 100%, and 150%). Thereafter, this BO stock solution was appropriately diluted to prepare a stock sample solution for TH, and the same standard addition procedure was applied.

[a] Mean ± standard deviation for five determinations.
[b] % Relative standard deviation.

Table S8: The penalty points of the proposed HPTLC method and reported HPLC methods according to the Analytical Eco-scale.

Reagents/ Instruments	Penalty Points (PPs)		
	HPTLC	Ion interaction based HPLC method [38]	Avidin binding based HPLC method [37]
**Chloroform**	4		
**Methanol**	6		
**Ammonia**	6		
**Phosphoric acid**		4	
**Salicylic acid**		4	
**Trifluoroacetic acid**			4
**Acetonitrile**			4
**HPTLC**	0		
**HPLC**		1	1
**Occupational hazards**	3	0	3
**Waste**	1	5	5
**PPs**	**20**	**14**	**17**
**Eco-scale score**	**80**	**86**	**83**

Table S9: Whiteness assessment of the proposed HPTLC methods and reported HPLC methods using RGB 12 algorithm.

Method name	R (%)	G (%)	B (%)	Whiteness (%)
**HPTLC**	87.5	90.8	98.3	92.2
**Ion interaction based HPLC method [38]**	82.5	90.0	89.8	87.4
**Avidin binding based HPLC method [37]**	90.0	84.2	74.6	82.9

Table S10: Detailed whiteness assessment and input data of the proposed HPTLC methods and reported HPLC methods using RGB 12 algorithm

RED PRINCIPLES (analytical performance)		R1: Scope of application	R2: LOD and LOQ			R3: Precision			R4: Accuracy		
	Method name	0-100	LOD	LOQ	0-100	RSD% (repeatability)	RSD% (reproducibility)	0-100	Relative error (%)	Recovery (%)	0-100
	HPTLC	60	0.33-0.58 microg/band	0.99-1.74	90	0.09-0.17	not applied	100	0.02- 0.56	99.65-100.36	100
	Ion interaction based HPLC method [38]	90	not	not	70	not	not applied	80	not	not	90
	Avidin binding based HPLC method [37]	100	not	not	80	0.9-12.5	NOT applied	90	not	not	90

GREEN PRINCIPLES (green chemistry)		G1: Toxicity of reagents (impact and biodegradation)		G2: Amount of reagents and waste			G3: Concumption of energy and other media	G4: Direct impacts (safety, use of animals and GMOs)			
	Method name	Total number of pictograms	0-100	Reagent consumption	Waste production	0-100	1-100	Occupational hazards	Safety of users (0-100)	Use of animals (0 if no, 1 if yes)	Use of GMO (0 if no, 1 if yes)
	HPTLC	**8**	70	rrelated with was	75ml/100runs	100	100	3	80	0	0
	Ion interaction based HPLC method [38]	**4**	90	correlated with w	5000ml/100runs	80	90	1	100	0	0
	Avidin binding based HPLC method [37]	**5**	80	correlated with w	5000ml/100runs	80	80	2	90	0	0

BLUE PRINCIPLES (practical side)		B1: Cost-efficiency		B2: Time-efficiency		B3: Requirements			B4: Operational simplicity		
	Method name	Total cost	0-100	Speed of analysis	0-100	Sample consumption	Sample consumption (0-100)	Other needs: advanced instruments, skills, facilities (0-100)	Miniaturization (0-100)	Integration and automation (0-100)	Portability (0-100)
	HPTLC	extremely low	100	extremely high	100	1ml/100 runs	100	100	100	90	90
	Ion interaction based HPLC method [38]	moderately low	90	slow	90	10ml/100 runs	90	95	90	90	80
	Avidin binding based HPLC method [37]	high	70	extremely slow	70	10ml/100 runs	90	80	80	70	70
