# Supplementary material for: Hydrolytic stress degradation study and concomitant HPTLC estimation of thioctic acid and biotin in their combined capsules: greenness, blueness and whiteness assessment
Source: BMC Chem. 2025 Oct 29;19(1):290. doi: 10.1186/s13065-025-01637-5 (PMC12574119; doi:10.1186/s13065-025-01637-5)
Supplement: Supplementary file 2 — Supplementary Material 2. [file 13065_2025_1637_MOESM2_ESM.pdf]

Supplementary File 2

AGREE input data

Analytical Greenness Report Sheets

HPTLC Method

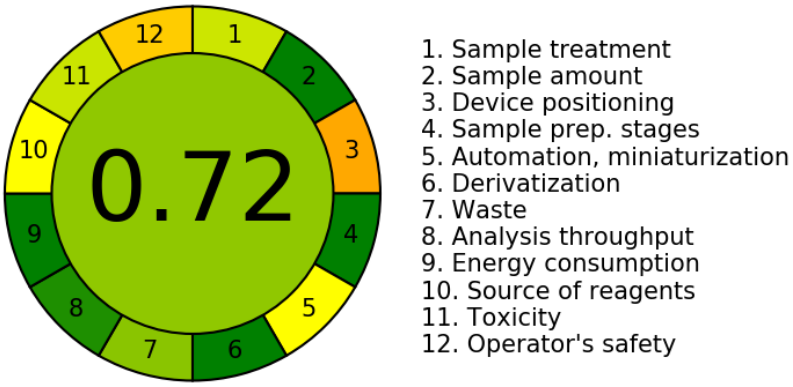

Criteria	Score	Weight
1. Direct analytical techniques should be applied to avoid sample treatment.	0.6	2
2. Minimal sample size and minimal number of samples are goals.	1.0	2
3. If possible, measurements should be performed in situ.	0.33	2
4. Integration of analytical processes and operations saves energy and reduces the use of reagents.	1.0	2
5. Automated and miniaturized methods should be selected.	0.5	2
6. Derivatization should be avoided.	1.0	2
7. Generation of a large volume of analytical waste should be avoided, and proper management of analytical waste should be provided.	0.73	2
8. Multi-analyte or multi-parameter methods are preferred versus methods using one analyte at a time.	0.94	2
9. The use of energy should be minimized.	1.0	2
10. Reagents obtained from renewable sources should be preferred.	0.5	2
11. Toxic reagents should be eliminated or replaced.	0.6	2
12. Operator's safety should be increased.	0.4	2

Ion interaction based HPLC method [38]

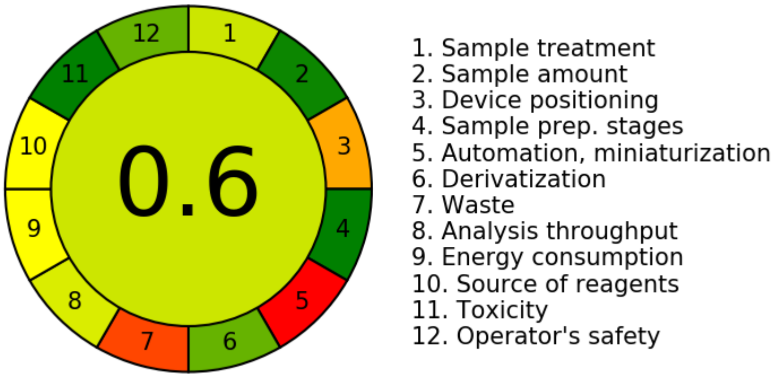

Criteria	Score	Weight
1. Direct analytical techniques should be applied to avoid sample treatment.	0.6	2
2. Minimal sample size and minimal number of samples are goals.	0.98	2
3. If possible, measurements should be performed in situ.	0.33	2
4. Integration of analytical processes and operations saves energy and reduces the use of reagents.	1.0	2
5. Automated and miniaturized methods should be selected.	0.0	2
6. Derivatization should be avoided.	0.8	2
7. Generation of a large volume of analytical waste should be avoided, and proper management of analytical waste should be provided.	0.14	2
8. Multi-analyte or multi-parameter methods are preferred versus methods using one analyte at a time.	0.57	2
9. The use of energy should be minimized.	0.5	2
10. Reagents obtained from renewable sources should be preferred.	0.5	2
11. Toxic reagents should be eliminated or replaced.	1.0	2
12. Operator's safety should be increased.	0.8	2

Avidin binding based HPLC method [37]

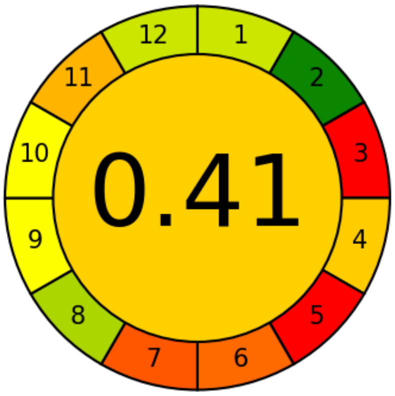

1. Sample treatment
2. Sample amount
3. Device positioning
4. Sample prep. stages
5. Automation, miniaturization
6. Derivatization
7. Waste
8. Analysis throughput
9. Energy consumption
10. Source of reagents
11. Toxicity
12. Operator's safety

Criteria	Score	Weight
1. Direct analytical techniques should be applied to avoid sample treatment.	0.6	2
2. Minimal sample size and minimal number of samples are goals.	0.98	2
3. If possible, measurements should be performed in situ.	0.0	2
4. Integration of analytical processes and operations saves energy and reduces the use of reagents.	0.4	2
5. Automated and miniaturized methods should be selected.	0.0	2
6. Derivatization should be avoided.	0.8	2
7. Generation of a large volume of analytical waste should be avoided, and proper management of analytical waste should be provided.	1.0	2
8. Multi-analyte or multi-parameter methods are preferred versus methods using one analyte at a time.	0.67	2
9. The use of energy should be minimized.	0.5	2
10. Reagents obtained from renewable sources should be preferred.	0.5	2
11. Toxic reagents should be eliminated or replaced.	0.35	2
12. Operator's safety should be increased.	0.6	2

MoGAPI Input Data

Modified Green Analytical Procedure Index

HPTLC Method

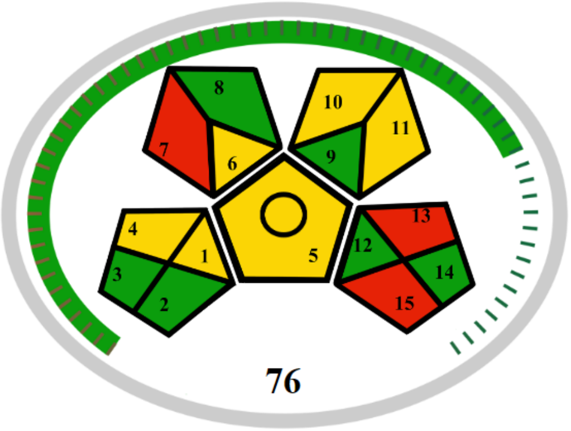

SAMPLE PREPARATION	
1 - Collection:	On-line or at-line
2 - Preservation:	None
3 - Transport:	None
4 - Storage:	Under normal conditions
5 - Type of method:	Simple procedures, e.g., filtration, decantation
6 - Scale of extraction:	Micro-extraction
7 - Solvents/reagents used:	Non-green solvents/reagents used
8 - Additional treatment:	None
REAGENT AND SOLVENTS	
9 - Amount:	< 10 mL (< 10 g)
10 - Health hazard:	Moderately toxic; could cause temporary incapacitation; NFPA = 2 or 3
11 - Safety hazard:	Highest NFPA flammability or instability score of 2 or 3, or a special hazard is used
INSTRUMENTATION	
12 - Energy:	≤0.1 kWh per sample
13 - Occupational hazard:	Emission of vapors to the atmosphere
14 - Waste:	< 1 mL (< 1 g)
15 - Waste treatment:	No treatment
16 - QUANTIFICATION:	Yes

Ion interaction based HPLC method [38]

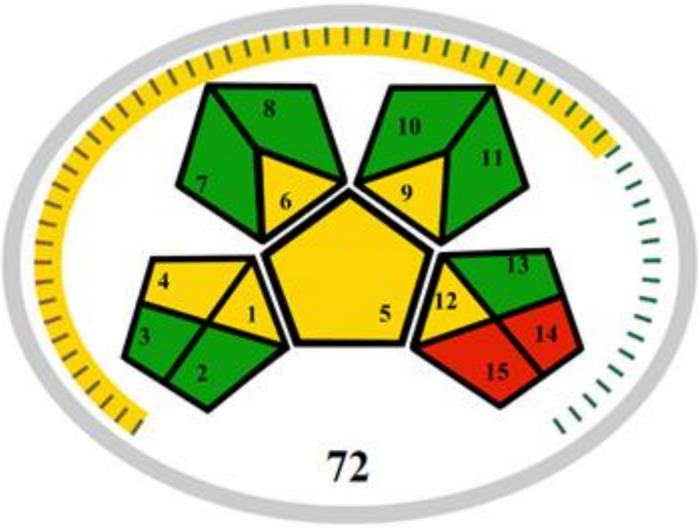

SAMPLE PREPARATION	
1 - Collection:	On-line or at-line
2 - Preservation:	None
3 - Transport:	None
4 - Storage:	Under normal conditions
5 - Type of method:	Simple procedures, e.g., filtration, decantation
6 - Scale of extraction:	Micro-extraction
7 - Solvents/reagents used:	Solvent-free methods
8 - Additional treatment:	None
REAGENT AND SOLVENTS	
9 - Amount:	10–100 mL (10–100 g)
10 - Health hazard:	Slightly toxic, slight irritant; NFPA health hazard score = 0 or 1
11 - Safety hazard:	Highest NFPA flammability or instability score of 0 or 1. No special hazards
INSTRUMENTATION	
12 - Energy:	≤1.5 kWh per sample
13 - Occupational hazard:	Hermetic sealing of analytical process
14 - Waste:	> 10 mL (> 10 g)
15 - Waste treatment:	No treatment
16 - QUANTIFICATION:	No

Avidin binding based HPLC method [37]

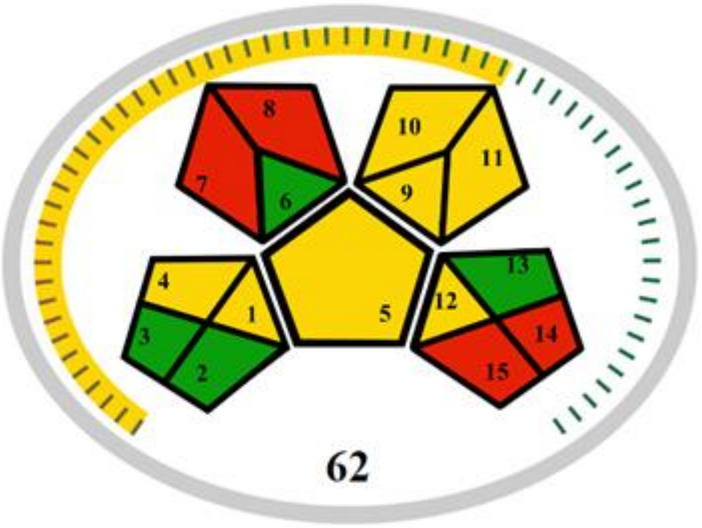

SAMPLE PREPARATION	
1 - Collection:	On-line or at-line
2 - Preservation:	None
3 - Transport:	None
4 - Storage:	Under normal conditions
5 - Type of method:	Simple procedures, e.g., filtration, decantation
6 - Scale of extraction:	Nano-extraction
7 - Solvents/reagents used:	Non-green solvents/reagents used
8 - Additional treatment:	Advanced treatments (derivatization, mineralization, etc.)
REAGENT AND SOLVENTS	
9 - Amount:	10–100 mL (10–100 g)
10 - Health hazard:	Moderately toxic; could cause temporary incapacitation; NFPA = 2 or 3
11 - Safety hazard:	Highest NFPA flammability or instability score of 2 or 3, or a special hazard is used
INSTRUMENTATION	
12 - Energy:	≤1.5 kWh per sample
13 - Occupational hazard:	Hermetic sealing of analytical process
14 - Waste:	> 10 mL (> 10 g)
15 - Waste treatment:	No treatment
16 - QUANTIFICATION:	No

BAGI Input Data

Blue Applicability Grade Index

HPTLC Method

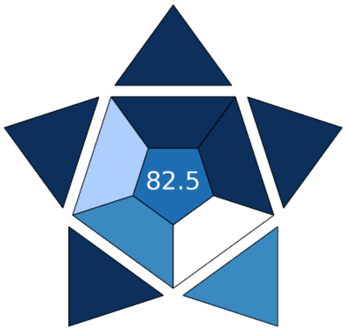

1. Type of analysis	Quantitative and confirmatory
2. Multi- or single-element analysis	Multi-element analysis for 2-5 compounds of the same chemical class
3. Analytical technique	Simple instrumentation available in most labs (UV, HPLC-UV, HPLC-DAD, UHPLC, FAAS, ETAAS, ICP-OES, GC-F
4. Simultaneous sample preparation	1
5. Sample preparation	Not required or on-site sample preparation if required
6. Samples per h	>10
7. Reagents and materials	Common commercially available reagents (methanol, acetonitrile, HNO3, nitrogen or other common gasses, e
8. Preconcentration	No preconcentration required. Required sensitivity and /or legislation criteria are met directly.
9. Degree of automation	Semi-automated with common devices (e.g. HPLC autosampler)
10. Amount of sample	<100 µL (or mg) bioanalytical samples; <10 mL (or g) food/environmental

Ion interaction based HPLC method [38]

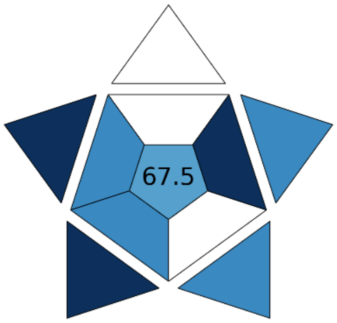

1. Type of analysis	Qualitative
2. Multi- or single-element analysis	Multi-element analysis for 6-15 compounds of the same chemical class or 2-15 compounds of different chen
3. Analytical technique	Simple instrumentation available in most labs (UV, HPLC-UV, HPLC-DAD, UHPLC, FAAS, ETAAS, ICP-OES, GC-F
4. Simultaneous sample preparation	1
5. Sample preparation	Not required or on-site sample preparation if required
6. Samples per h	≤1
7. Reagents and materials	Common commercially available reagents (methanol, acetonitrile, HNO3, nitrogen or other common gasses, e
8. Preconcentration	No preconcentration required. Required sensitivity and /or legislation criteria are met directly.
9. Degree of automation	Semi-automated with common devices (e.g. HPLC autosampler)
10. Amount of sample	100-500 μL (or mg) bioanalytical samples; 10.1-50 mL (or g) food/environmental

Avidin binding based HPLC method [37]

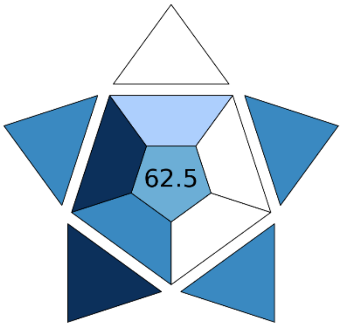

1. Type of analysis	Screening
2. Multi- or single-element analysis	Multi-element analysis for > 15 compounds
3. Analytical technique	Simple instrumentation available in most labs (UV, HPLC-UV, HPLC-DAD, UHPLC, FAAS, ETAAS, ICP-OES, GC-F
4. Simultaneous sample preparation	1
5. Sample preparation	Multi step sample preparation required (e.g. LLE, SPE and/or derivatization)
6. Samples per h	≤1
7. Reagents and materials	Commercially available reagents not common in QC labs (derivatization reagents, SPE cartridges, SPME fibers
8. Preconcentration	No preconcentration required. Required sensitivity and /or legislation criteria are met directly.
9. Degree of automation	Semi-automated with common devices (e.g. HPLC autosampler)
10. Amount of sample	100-500 µL (or mg) bioanalytical samples; 10.1-50 mL (or g) food/environmental
